# Candidate transdiagnostic processes linking potentially traumatic experiences to psychopathology, mental well-being and resilience in emerging adults

**DOI:** 10.1186/s12888-026-08302-8

**Published:** 2026-06-22

**Authors:** Caroline Cohrdes, Vera Birgel, Sonja Entringer, Michael Rapp, Mira Tschorn, Heike Hölling

**Affiliations:** 1 Mental Health Research Unit, Department of Epidemiology and Health Monitoring, Robert Koch Institute, Nordufer 20, 13353 Berlin, Germany; 2 German Centre for Mental Health (DZPG), Berlin, Germany; 3 Department of Social and Preventive Medicine, University of Potsdam, Potsdam, Germany; 4 Institute of Medical Psychology, Charité Universitätsmedizin Berlin, Berlin, Germany

**Keywords:** Trauma, Candidate transdiagnostic process variables, Social exclusion, Mental health, Emerging adults, Indirect association models

## Abstract

**Background:**

Potentially traumatic events (PTEs), including socially contextualized adversities such as exclusion and discrimination, are common at the population level and associated with diverse mental health outcomes. Transdiagnostic process variables may help characterize how PTE indicators and different mental health outcomes are interrelated beyond disorder-specific frameworks.

**Methods:**

We analyzed data from a population-based sample of 3,051 emerging adults to examine whether theoretically informed transdiagnostic psychological processes were statistically positioned between PTE indicators and mental health outcomes, including psychopathology, positive mental health, and resilience. Guided by transdiagnostic and dimensional frameworks, we used structural equation models to estimate cross-sectional indirect associations.

**Results:**

Three higher-order transdiagnostic factors—cognitive-focused, emotional-focused, and social-focused processes—were identified. Emotional-focused processes accounted for the largest proportion of cross-sectional indirect associations across mental health outcomes. Among the examined PTEs, social exclusion showed the strongest and most consistent associations with mental health. Overall, the models accounted for more variance in internalizing symptoms than in externalizing symptoms or positive mental health outcomes.

**Conclusions:**

These findings highlight the relevance of candidate transdiagnostic process variables for understanding mental health following exposure to adversity. By incorporating positive mental health and resilience outcomes, the results underscore the potential of targeting shared psychological processes—particularly emotional-focused mechanisms—for assessment, prevention, and early intervention in psychiatric practice.

**Clinical trial number:**

Not applicable.

**Supplementary Information:**

The online version contains supplementary material available at 10.1186/s12888-026-08302-8.

## Introduction

*Potentially traumatic events* (PTEs) are common at the population level, with prevalence estimates ranging from approximately one in four young adults for narrowly defined traumatic events to up to 70–80% when broader definitions are applied [1–3]. Although PTE exposure is associated with increased risk of mental health problems, including post-traumatic stress disorder (PTSD), most exposed individuals do not develop a specific disorder, highlighting substantial heterogeneity in outcomes [4, 5]. PTEs encompass a wide range of experiences, including adverse childhood experiences (e.g., abuse and neglect), exposure to violence or serious accidents, bereavement, and natural disasters, experienced directly or as a witness [4, 5]. Guided by the *Physical and Social Trauma* (PAST) [6] framework, potentially traumatic events are increasingly conceptualized as a spectrum that also includes threats to fundamental social needs (e.g., acceptance, belonging) and their violation through, for instance, bullying or social exclusion, thereby broadening the concept of trauma to include experiences such as social exclusion. The consequences of PTEs extend beyond individual psychopathology, affecting physical health, social functioning, and economic participation, and contributing to population-level mental health burden and social costs [7–9]. From a social psychiatric and epidemiological perspective, PTEs therefore represent key public health exposures that contribute to mental health inequalities and long-term societal costs such as reduced productivity, enhanced hospitalization and healthcare costs, delinquency and incarceration [7, 10, 11]. PTEs are socially patterned exposures shaped by structural inequalities, making it essential to understand the psychological processes through which they contribute to population-level mental health disparities.

*Emerging adulthood* is a particularly sensitive period, characterized by both high exposure to PTEs and increased vulnerability to adverse mental health outcomes [12, 13]. This is particularly relevant given that emerging adulthood (i.e., the period from late adolescence to the late twenties living in industrialized societies) [14], is a critical developmental phase involving complex changes and challenges in various life domains, which are linked to increased demands on coping and adaptation skills [14, 15]. The majority of trauma-related disorders (e.g., PTSD, depression, panic disorder) manifest during this phase with a median age of onset at 20.5 years [16]. Confronting the diverse changes and challenges during this time, however, also provides opportunities for growth, leading to high heterogeneity in developmental paths and mental health outcomes. Accordingly, the risk of developing manifest mental or physical health conditions following exposure to PTEs has been shown to vary significantly depending on the type and severity of the exposure, as well as the individual’s resources and support they receive from others [12]. From a population health perspective, emerging adulthood represents a critical window for prevention, as early intervention may prevent the consolidation of mental disorders and promote long-term resilience [15, 17].

To explain heterogeneity in mental health outcomes following PTE exposure, increasing attention has been directed toward transdiagnostic mechanisms—psychological processes that cut across diagnostic categories [18]. In this context, the term *transdiagnostic* is used to describe processes underlying the development or maintenance of a wide range of mental health conditions [19]. These processes may include cognitive mechanisms, such as negative appraisals, executive functioning, and cognitive reappraisal; emotional mechanisms, such as emotion regulation, suppression, and negative affect; and social mechanisms, such as interpersonal insecurity, perceived social threat, and support-seeking behavior. These mechanisms help to identify possible explanations for an increased risk of multiple mental health disorders in response to stress and trauma, transcending traditional diagnostic boundaries [18]. Transdiagnostic and dimensional approaches align closely with social psychiatric and epidemiological perspectives by conceptualizing mental health on a continuum rather than as discrete categories. Over the past 10 to 15 years, the transdiagnostic perspective has evolved significantly and become established in mental health research and practice. Frameworks such as the Hierarchical Taxonomy of Psychopathology (HiTOP) [20] and the Research Domain Criteria (RDoC) [21] provide empirically grounded structures for organizing transdiagnostic processes relevant to population mental health. They emphasize the dimensional approach along a continuum of symptom severity, based on either empirical patterns of symptom co-occurrence [20] or on a combination of observed behavior, neurodevelopmental and environmental factors [21]. Both the HiTOP and RDoC have gathered empirical evidence in support of their theoretical and structural assumptions [22, 23]. In the present study, the term transdiagnostic is used in a framework-guided and descriptive sense. The selected process indicators were derived from transdiagnostic and dimensional frameworks and were examined across multiple mental health outcome domains rather than within a single diagnostic category; therefore, we refer to them as candidate transdiagnostic process variables rather than established transdiagnostic mechanisms.

Although prior research has varied in how transdiagnostic factors are operationalized, accumulating evidence suggests that such processes are associated with both PTE exposure and mental health outcomes and may account for shared variance between them [17, 24, 25]. Although transdiagnostic frameworks emphasize the interconnected nature of psychological functioning, many models organize transdiagnostic processes into broad domains reflecting cognitive, emotional, and social functioning [24, 31, 36–39]. Cognitive processes refer to how individuals appraise, interpret, and respond to experiences, including cognitive flexibility, problem solving, and reappraisal. Emotional processes encompass affective responses and emotion-regulation tendencies, such as negative affect and maladaptive coping. Social processes involve the interpersonal context in which emotions and behavior are regulated, including support-seeking, social connectedness, and the management of emotional expression in social interactions. These domains are not mutually exclusive, and many psychological processes involve cognitive, emotional, and social components simultaneously. Nevertheless, they provide a useful conceptual framework for organizing candidate transdiagnostic process variables and examining their associations with mental health outcomes across diagnostic boundaries.

From an epidemiological standpoint, these mechanisms represent candidate explanatory variables linking social exposures to mental health outcomes. In addition to such mediating psychological processes, interpersonal resources may shape the strength of associations between PTEs and mental health. Social support, for example, has consistently been identified as a protective factor in trauma response and recovery, as it may provide emotional, informational, and practical resources that help individuals manage adversity [26–28]. In the present study, perceived social support was therefore not conceptualized as one of the transdiagnostic process variables, but as a contextual interpersonal resource that may moderate the direct associations between PTEs and mental health outcomes [29].

Traumatic experience, particularly during childhood and adolescence, disrupts the development and functioning of neurobiological, behavioral and psychological systems [30, 31]. However, there is still scarce evidence on which processes are most helpful in promoting mental health and preventing a range of mental disorders or spectra after PTE exposure [32]. There are also only a few studies that included positive mental health outcomes (e.g., mental well-being) in addition to psychopathology [25]. Previous studies with a predominant focus on psychopathological outcomes have suggested that the associations between PTEs and mental health are best explained by underlying transdiagnostic mechanisms involving cognitive processes (e.g., negative appraisals or beliefs), emotional processes (e.g., regulation, awareness), social processes (e.g., biased attention or sensitivity to social cues), and epigenetic (e.g., immune and inflammatory response) or neurobiological (e.g., accelerated biological ageing) modifications [24, 25, 33, 34]. An example of a social explanatory process is that individuals with a history of trauma develop a bias in processing social information in a way that they interpret and experience social cues as more threatening or have higher expectations about negative attitudes from others towards themselves [27], or they tend to exhibit heightened emotional responses to potential threats and difficulty in disengaging from negative emotions [27, 35]. This is considered dysfunctional emotional processing, which in turn contributes to psychopathology [24].

However, it is somewhat difficult to categorize such processes into distinct higher-order factors due to interconnectivity and overlap, which has led to heterogeneity thus far. For example, self-regulation involves all three of the aforementioned psychological processes (cognitive, emotional and social), making it difficult to assign to one category only [36–38]. The cognitive aspect of self-regulation interacts with emotional and social processing such as in reframing a situation to enable modifying emotions and altering their (negative) impact; the emotional aspect concerns, among other things, the ability to evaluate and modify emotions (based on cognitive reframing); self-regulation embedded in social contexts allows persons to align their behavior, such as emotional expression, with social norms and expectations [39]. However, for the practical implementation and development of support measures, it is often necessary to define and focus on a distinct factor or process. Furthermore, the use of strategies to cope with stress and regulate emotions [30], as well as flexibility in the selection and application of such strategies [40, 41], have been pointed out as key transdiagnostic factors in preventing the emergence of mental illness, so far. As prior studies predominantly investigated transdiagnostic mechanisms individually, it remains difficult to derive robust, generalizable findings [25, 42]. Moreover, there is a lack of information on how different types of trauma (e.g., physical, emotional, social) relate to positive and psychopathological mental health conditions [43].

The overall aim of this study was to extend prior research by examining a comprehensive set of transdiagnostic process variables alongside multiple types of PTEs in a population-based sample. Based on the transdiagnostic model proposed by McLaughlin et al. 2020 [24], this research investigated how broad domains of psychological processing are positioned within the cross-sectional association structure linking PTEs and mental health outcomes. Because the present analyses are based on nonexperimental cross-sectional data, the identified process variables cannot be interpreted as temporal or causal mediators. Instead, we conceptualize them as theoretically informed associational variables that may be statistically positioned between PTE indicators and mental health outcomes. This approach allows us to examine how PTE indicators, transdiagnostic process variables, and mental health outcomes are interrelated, while treating the findings as an initial step toward future longitudinal or experimental tests of mediation. Considering high heterogeneity, we aimed to identify empirically supported higher-order factors that categorize and differentiate such processes. In addition to psychopathological outcomes, we also included positive mental health and resilience outcomes, which have been neglected so far, to examine the potential of results not only for prevention but also for mental health promotion.

Specifically, we aimed to (1) identify empirically supported higher-order transdiagnostic process factors representing psychological processes (i.e., cognitive, emotional, social); (2) examine whether these higher-order factors are statistically positioned between PTE indicators and mental health outcomes in cross-sectional indirect association models; (3) examine whether these association patterns differ by various types of PTEs, including socially contextualized adversities, and (4) exploratorily examine whether perceived social support moderates the direct associations between PTEs and mental health outcomes.

Based on transdiagnostic and dimensional frameworks, we expected that cognitive-, emotional-, and social-focused process factors would be associated with both PTE indicators and mental health outcomes. We further expected emotional-focused processes, including negative affect, suppression, and maladaptive coping, to show the strongest indirect associations, particularly for internalizing symptoms. Given the social nature of exclusion and discrimination, we expected socially contextualized PTE indicators to show particularly consistent associations with emotional- and social-related process factors. The moderation by perceived social support was examined exploratorily.

## Methods

### Sample and procedure

The study on the mental health of young adults in Germany (JEPSY), is composed of former study participants (i.e., a nationally representative study on the health of children and adolescents), during their transition to adulthood (aged 16–25, as of 1 March, 2024).

The JEPSY study is a follow-up study of former participants of the German Health Interview and Examination Survey for Children and Adolescents (KiGGS), conducted by the Robert Koch Institute. Eligible participants were individuals who had participated in KiGGS Wave 2 between 2014 and 2017 and had consented to be re-contacted. KiGGS Wave 2 included both a longitudinal cohort sample, consisting of participants who had already taken part in previous KiGGS waves, and a newly recruited cross-sectional sample, designed to ensure population representativeness at the time of KiGGS Wave 2. Participants from both samples were invited to register on the Health online platform and subsequently participate in JEPSY.

Individuals from both the national cohort sample and cross-sectional samples of Wave 2 (2014–2017), who gave informed consent to be re-contacted, were invited to register on the Health online platform and subsequently participate in JEPSY. The two-fold recruitment procedure covered a period from February to July 2024 and involved: (1) A postal invitation (including two reminders) to create a Health platform account via a QR code or URL and (2) an email invitation (including two reminders) to complete the JEPSY study online. By registering, participants agreed to the storage of their contact details and to receiving invitations to participate in studies. Study participation required informed consent. Participants received compensation in the form of universal vouchers: 10 EUR upon registration and another 5 EUR upon completion of the study. A more detailed description of the study methodology can be found in Lange et al. [44].

Of the 11,815 eligible participants invited to take part in the JEPSY study (4,401 from the national cohort; 7,414 from the cross-sectional sample of Wave 2; 50.5% female), 4,451 registered on the Health platform (1,822 from the national cohort; 2,629 from the cross-sectional sample of Wave 2; 62.5% female). Of the registered young adults, a total of 3,063 (65.9% female) participated in the JEPY study. Following data quality and plausibility analyses, 12 participants were excluded due to at least two out of four conspicuous parameters indicating careless responding (i.e., straightlining, intra-individual response variability, Mahalanobis distance, psychometric synonym index) resulting in a final sample of 3,051 investigated JEPSY participants (66.0% female, mean age = 21.08, SD = 2.68). Most participants had a high level of education (German university entrance qualification; 61.1%), while nearly one-third (27.8%) had a moderate and 1.7% had a lower level or no educational degree, 9.3% were still in school.

Attrition analysis revealed certain factors influencing registration on the platform and participation in the JEPSY study, such as the sex assigned at birth and the parental socioeconomic status [44]. To reduce potential selection bias, we applied sample weights to adjust the composition of the JEPSY study sample to that of the original nationally representative Wave 2 adolescent sample.

### Measures

Supplementary Table S1 provides an overview of the outcome and potentially traumatic event measures, including full instrument names, abbreviations, item sources, response formats, and scoring. Supplementary Table S2 provides detailed information on the transdiagnostic process indicators, including source instruments, number of items, coding direction, and assignment to the cognitive-, emotional-, and social-focused factors.

#### Mental health outcomes

To capture mental health on a broad scale, we have included both positive mental health and resilience outcomes as well as symptoms of psychopathology. We assessed general life satisfaction (L1) [45] and meaning of life (first item of the Meaning in Life Questionnaire, MLQ) [46] with one item each answered on a scale from 0 (not at all) to 10 (completely) as well as psychological resilience via the 2-item version of the Connor-Davidson Resilience Scale (CD-RISC-2) [47]. The responses to the CD-RISC-2 were averaged. Participants’ psychopathology was assessed using a subset of items from the Diagnostic and Statistical Manual of Mental Disorders, Fifth Edition, Text Revision (DSM-5-TR) [48], the Personality Inventory for DSM-5 (PID-5) [49], and the Patient Health Questionnaire-4 (PHQ-4) [50]. The initial selection of items was guided by expert consensus within the Minimum Data Set (MDS) working group of the German Center for Mental Health (DZPG) [51]. Based on subsequent factor-analytic validation, the best-fitting items were summarized into two externalizing (personality traits, substance use) and one internalizing factor. The first externalizing factor is composed of four DSM-5-TR items for alcohol, tobacco, and non-prescription drug use, the second externalizing factor encompasses five PID-5 items related to disinhibition, including risk-taking and impulsivity [52]. The internalizing factor is constituted of ten PHQ-4 and DSM-5-TR items for anxiety and depressive symptoms, such as nervousness and anhedonia [52]. The found factorial structure has been validated in another representative population sample [52]. For each respondent, these three factor scores were min–max-standardized to a 0–1 range, with higher values indicating greater symptom severity.

#### Potentially traumatic events

As indications for potentially traumatic events during childhood and adolescence we used the Childhood Trauma Screener (CTS) [53] including 5 items answered on a 5-point rating scale from (0) never to [4] very often to measure emotional and physical neglect, as well as sexual, emotional and physical abuse; the Primary Care Posttraumatic Stress Disorder Screen for DSM-5 (PC-PTSD-5) [54] with binary response categories (yes/no) referring to lifetime experience of a frightening, horrible, or traumatic event (e.g., war or seeing someone be killed or seriously injured) – in the following referred to as “PTSD-related event”; the experience of critical life events (CLEs) from the areas of social, occupational, or health (e.g., separation/divorce, job loss, severe illness) in accordance with the Social Readjustment Rating Scale [55] within the past 12 months. To also account for the harmful effects that involve social threats [6, 56], we considered reports of social discrimination and exclusion as indications of potentially traumatic experience via two items developed by Tschorn et al. (2025) [51] for a Minimum Data Set embedded within DZPG. Both items were answered with reference to the last 30 days on a visual analog scale ranging from 0 to 100. For the present analyses we used the CTS as well as social discrimination and exclusion single item values, the cut-off of 3 out of five questions across the five PC-PTSD-5 symptoms to build categories of 1 (PTSD-related event) and 0 (no PTSD-related event), and categories of 1 (yes) and 0 (no) for at least one reported critical life event within the three areas of social, occupational, and health events.

#### Transdiagnostic process indicators

To investigate candidate transdiagnostic process variables that may be statistically positioned between PTE indicators and mental health outcomes, we included a selection of items from the RDoC Matrix [23] and from the list of HiTOP-friendly measures [57]. The selection was based on a consensus process for the development of the MDS within the DZPG. More precisely, we included emotion regulation composed of cognitive reappraisal and expressive suppression subscales (Emotion Regulation Questionnaire, ERQ) [58], eight coping strategies consisting of two averaged items each (e.g., social support coping, wishful thinking) from the Short Adult Coping Scale (SACS-16) [59], 3 summarized items to a total score for executive functioning (DSM-5-TR [48], Behavior Rating Inventory of Executive Function (BRIEF-A) [60]), 2 negative affect items combined to a total score (Positive and Negative Affect Schedule, PANAS) [61], 4 summarized items to measure uncertainty in social contact (Interpersonal Sensitivity Measure, ISPM) [62], and single items assessing empathy (Level of Personality Functioning Scale-Brief Form, LPFS-BF) [63], and ability to maintain friendships (World Health Organization Disability Assessment Schedule 2.0, WHO-DAS 2.0) [64]. Consistent with transdiagnostic frameworks and prior theoretical models [24, 36–39], the selected indicators were expected to broadly reflect cognitive, emotional, and social domains of functioning, while acknowledging that some indicators may contain elements of more than one domain. To ensure consistent interpretation across variables, all items were recoded where necessary so that higher values uniformly indicate more favorable characteristics in terms of cognitive, emotional or social functioning—such as more frequent use of adaptive coping strategies, less emotional suppression, or lower social uncertainty. Details on the indicators used to derive the cognitive-, emotional-, and social-focused process factors are provided in Supplementary Table S2.

#### Perceived social support

Since perceptions of the availability and quality of social relations play a crucial role in trauma response and recovery, we considered the perceived level of social support as a potential moderator in our analyses, measured with the 3-item Oslo Social Support Scale (OSS-3) [65]. Item responses were summed to a total score ranging from 3 to 14, with higher scores indicating stronger perceived social support.

### Statistical analyses

Statistical analyses were performed using R version 4.2.2 [66] and RStudio (04.0.735). Psychometric analyses were carried out with the *psych* package [67], structural equation modelling with *lavaan* [68], and survey weighting via *survey* [69].

Before factor extraction, the Kaiser–Meyer–Olkin measure and Bartlett’s test of sphericity were used to assess suitability. An exploratory factor analysis (principal-axis, oblimin rotation) was run; the number of factors was chosen based on parallel analysis, scree-plot inspection and the Kaiser criterion. The emergent structure was evaluated in a confirmatory factor analysis, which was estimated using robust maximum likelihood (MLR) and full-information maximum likelihood for missing data. Item-level missingness was low across all variables used in the factor analysis, ranging from 0% to 0.7%. Model fit was judged acceptable when CFI / TLI ≥ 0.90, and RMSEA/SRMR ≤ 0.08. The resulting latent constructs were exported as Bartlett factor scores and used as candidate process variables in subsequent cross-sectional indirect association models.

To address research objectives 2 and 3, we specified separate structural equation models (SEMs) for the following six mental health outcomes: psychological resilience, satisfaction with life, meaning of life, and three min–max standardized symptom dimensions (internalizing, externalizing–personality, externalizing–substance). Predictors included 11 indicators for PTEs including childhood trauma, posttraumatic stress symptoms, critical life events (related to job, health, and social factors), and perceived social exclusion and discrimination. The final latent transdiagnostic factors served as intervening process variables. Age, sex assigned at birth, general subjective health, and education were included as covariates. General subjective health was included as a covariate and assessed using the self-rated health item from the Minimum European Health Module (MEHM): “How is your health in general?” Response options were “very good”, “good”, “fair”, “bad”, and “very bad”. To test for conditional effects of social support, each SEM included interaction terms between the predictors and the perceived level of social support. These interaction terms were added as moderators on the direct paths from the predictors to the respective outcomes (path c’). All models included direct associations from each predictor to the respective outcomes, as well as indirect associations via latent mediators (a–b–c structure). These indirect paths were interpreted as cross-sectional association patterns rather than evidence of temporal or causal mediation. Total associations were defined as the sum of the direct and the associated indirect paths. The models were estimated using nonparametric bootstrapping with 2,000 resamples and robust maximum likelihood estimation (MLR).

Calibrated design weights were applied for descriptive analyses to reduce selection bias and improve alignment with the original KiGGS Wave 2 adolescent sample. The SEM analyses were conducted without sampling weights because combining calibrated survey weights, bootstrapped indirect paths, and a highly parameterized SEM including multiple interaction terms is methodologically complex and can lead to unstable estimates. We therefore interpreted the SEM parameters as model-based association estimates within the analytic sample rather than as fully population-representative effect estimates.

We addressed multiplicity arising from the large number of simultaneously tested structural paths by controlling the false discovery rate (FDR) using the Benjamini–Hochberg (BH) procedure. Specifically, we computed BH-adjusted p-values across all SEMs, pooled over outcomes, separately by effect type: (i) all direct effects, (ii) all indirect effects, and (iii) all moderation effects. This approach preserves interpretability by correcting within conceptually homogeneous families of tests while accounting for the extensive parallel testing across outcomes. Because the SEMs included multiple PTE indicators, process variables, outcomes, and interaction terms, the analyses involved a large number of simultaneously estimated paths. The BH correction reduces, but does not eliminate, the possibility of chance findings. Accordingly, individual significant paths were interpreted cautiously, and emphasis was placed on recurring patterns across outcomes and process factors rather than isolated associations. Total effects were reported with bootstrap-based 95% confidence intervals but were not included in the FDR correction, as they are deterministic functions of the direct and indirect effects and therefore do not constitute an additional independent family of hypothesis tests. Adjusted p-values are reported alongside unadjusted p-values, and statistical significance was evaluated at an FDR q-value of 0.05.

**Fig. 1:**
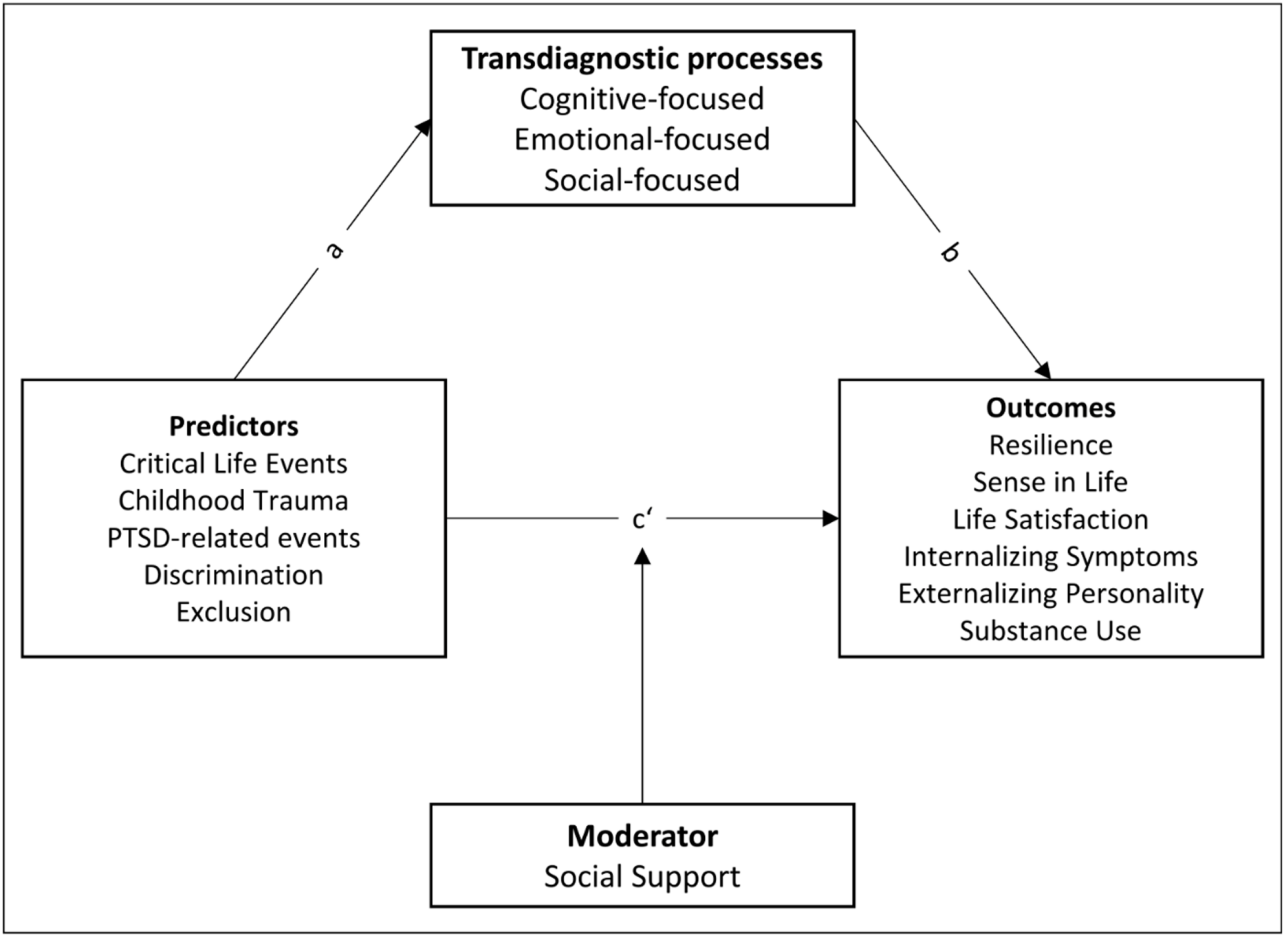
Conceptual indirect association model illustrating direct and indirect associations between potentially traumatic event indicators and mental health outcomes via transdiagnostic process variables, while considering perceived social support as a moderator and age, sex assigned at birth, general subjective health, and educational level as covariates (not shown). The total association between potentially traumatic experiences (X) and outcomes (Y) is decomposed into a direct association (c′) and an indirect association (a × b) via transdiagnostic process variables (M), including cognitive-, emotional-, and social-focused processes

Figure 1 shows the composition of the theoretical model examined via SEM.

To facilitate interpretation of the magnitude of specific indirect effects, we additionally calculated the standardized indirect effects (SIE) with the R package *manymome* [70], multiplying the standardized regression coefficient (Beta) by the ratio of the predictor’s standard deviation (SDx) to the outcome’s standard deviation (SDy) [71]. Predictors originally measured on a 0–100 scale (i.e., perceived discrimination and exclusion) were min–max normalized to a 0–1 range to avoid inflation of effect sizes, as other predictors had substantially narrower scales (e.g., 0–1 or 0–2). The standardized indirect effect ranges from − 1 to 1, with higher values indicating larger effects. Model assumptions—including multicollinearity, residual distribution and heteroskedasticity—were checked prior to interpretation (see Supplementary materials S3).

## Results

To examine whether the included candidate process variables reflect distinct transdiagnostic processes, an exploratory factor analysis (EFA) using oblimin rotation was conducted first. The factorability of the data was confirmed by a high Kaiser-Meyer-Olkin measure (KMO = 0.84) and a highly significant Bartlett’s test of sphericity (χ² = 11,447, *p* < .001), indicating sufficient intercorrelations among the variables. The parallel analysis and scree plot supported extracting three factors. These three factors had eigenvalues of 2.16, 1.99, and 1.79, respectively, explaining a total of 39.6% of the variance (14.4%, 13.2% and 11.9% each). Two variables were excluded due to poor and ambiguous loadings: empathy (loadings of 0.07 for factor 1, 0.21 for factor 2, and 0.24 for factor 3) and executive functioning (loadings of 0.28 for factor 1, − 0.02 for factor 2, and 0.38 for factor 3).

The resulting structure suggested a meaningful grouping of three factors labeled cognitive-focused, emotional-focused, and social-focused transdiagnostic processing. Factor 1, labeled “Cognitive-focused processes”, included items reflecting cognitive control and flexible coping strategies, such as cognitive reappraisal, problem solving, persistence, coping flexibility, and proactive coping. Factor 2, labeled “Emotional-focused processes” captured maladaptive emotional coping strategies and social insecurities, including suppression, wishful thinking, negative affect, social withdrawal, and uncertainty in social interactions. “Social-focused processes”, representing the third factor, reflected adaptive interpersonal processes, such as seeking emotional and instrumental support, and low levels of suppression. Table 1 summarizes the results from EFA.

**Table 1:** Factor loadings (> 0.30), communalities & uniqueness

Variable	F1	F2	F3	Communality	Uniqueness
Emotion regulation: cognitive reappraisal	0.44			0.21	0.79
Proactive coping	0.55			0.28	0.72
Active coping: perseverance	0.57			0.48	0.52
Active coping: problem solving	0.69			0.53	0.47
Coping flexibility	0.75			0.60	0.40
Ability to maintain friendships		0.39		0.23	0.77
Uncertainty in social contact		0.52		0.30	0.70
Negative affect		0.54		0.37	0.63
Avoidance: repression		0.57		0.36	0.64
Avoidance: wishful thinking		0.66		0.41	0.59
Emotion regulation: expressive suppression			0.57	0.42	0.58
Instrumental support-seeking			0.68	0.53	0.47
Emotional support-seeking			0.89	0.78	0.22

Notes*.* F1 = cognitive-focused factor, F2 = emotional-focused factor, F3 = social-focused factor

Second, a confirmatory factor analysis (CFA) was conducted using robust maximum likelihood estimation (MLR) to confirm the factor structure identified through EFA.

The final measurement model was selected after a series of exploratory and confirmatory factor-analytic specifications guided by established psychometric criteria, including factorability, parallel analysis, scree-plot inspection, the Kaiser criterion, standardized factor loadings, weak or ambiguous loadings, theoretical interpretability, and standard fit indices. Because these analyses were conducted to derive a parsimonious and interpretable measurement model rather than to test competing substantive hypotheses, only the initial and final CFA specifications are reported in detail.

**Fig. 2:**
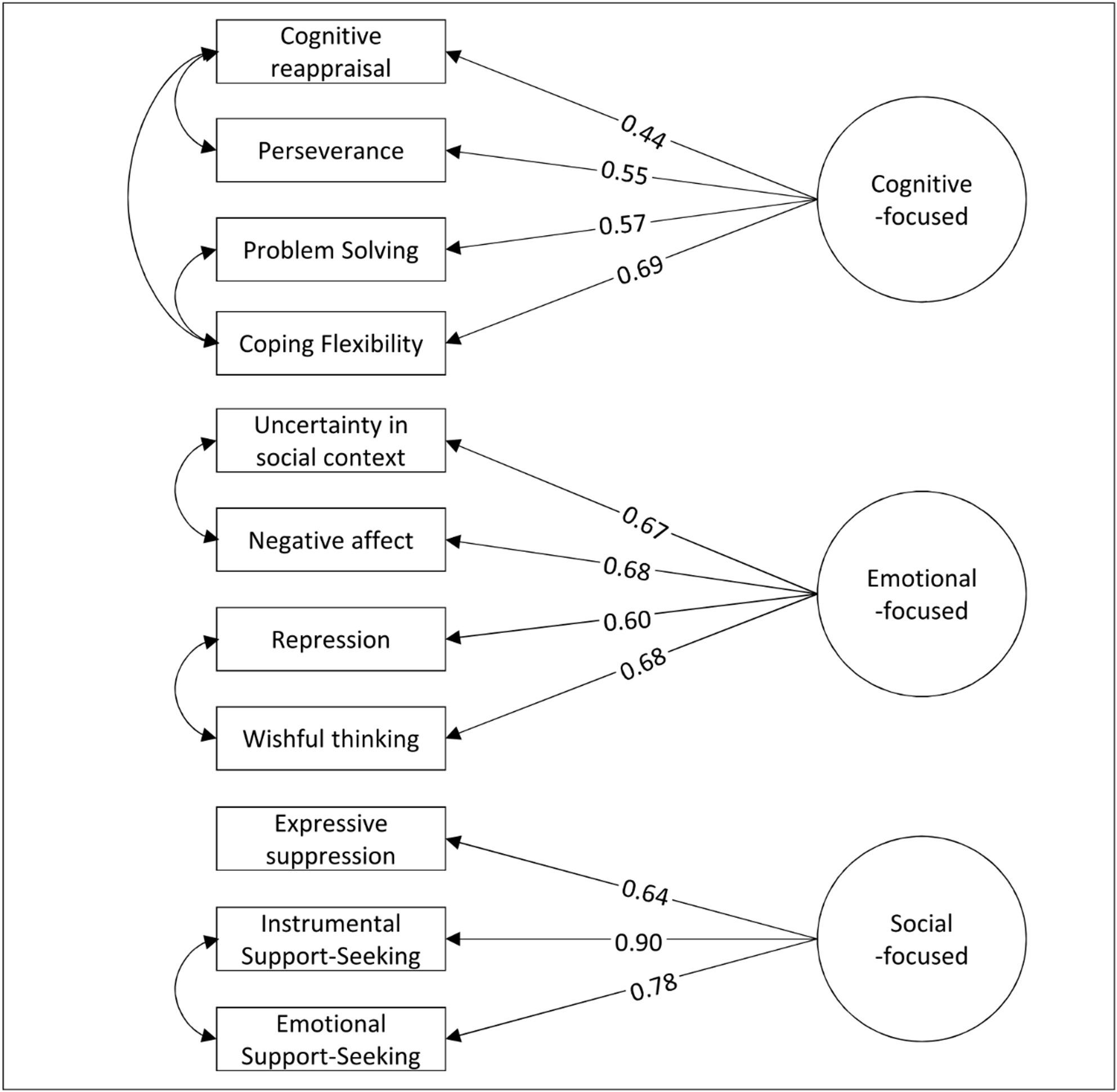
Results of Confirmatory Factor Analysis suggesting 11 suitable predictors that loaded onto three higher-order cognitive-, emotional, and social-focused factors. Empathy, executive functioning, proactive coping, and difficulties maintaining friendships were excluded due to low and/or ambiguous factor loadings

The initial model, which retained all EFA variables, showed only moderate fit. Although *RMSEA* and *SRMR* were within acceptable thresholds, *CFI* and *TLI* fell slightly below the cutoffs (*CFI* = 0.886, *TLI* = 0.865). Two indicators, proactive coping and difficulties maintaining friendships, had weak loadings and were therefore excluded from subsequent models. Indicators were excluded when they showed weak or ambiguous empirical relations with the intended higher-order factors and would therefore have reduced factorial clarity. Conceptually, empathy, executive functioning, proactive coping, and friendship maintenance remain relevant to transdiagnostic models; however, in the present item set they did not form sufficiently distinct indicators of the three empirically derived factors. Model modification indices suggested several residual correlations within latent factors, which were theoretically plausible and were subsequently included. Specifically, within the factor cognitive-focused processes, residual correlations were allowed between cognitive reappraisal and coping perseverance, as well as between coping flexibility and both problem-solving and cognitive reappraisal, reflecting their conceptual overlap in adaptive cognitive coping strategies. Within the factor emotional-focused processes, residual covariances were added between coping repression and wishful thinking, and between negative affect and social insecurity. Finally, within the social-focused processes factor, a covariance between emotional and instrumental support seeking was specified, given their shared reliance on external support systems. Residual covariances were only added within the same latent factor and when they were theoretically plausible due to overlapping item content or shared method features. They were not added across latent factors or based solely on statistical criteria. The final CFA model consisted of 11 indicators across three factors and demonstrated good model fit with *CFI* = 0.952, *TLI* = 0.912, *RMSEA* = 0.068 [90% CI: 0.063–0.074], and *SRMR* = 0.038 (see Fig. 2; Table 2).

All factor loadings were statistically significant (*p* < .001) and exceeded 0.44, with explained variances (R²) ranging from 0.195 to 0.804. Factor correlations were moderate, indicating conceptual overlap while supporting distinct latent constructs (*r* = .51 between cognitive- and emotional-focused processes, *r* = .45 between cognitive- and social-focused processes, and *r* = .25 between emotional- and social-focused processes). The final factors comprised (1) indicators related to cognitive control and reappraisal, including cognitive flexibility, persistence, problem-solving, and reappraisal (“Cognitive-focused processes”), (2) items representing maladaptive coping and affective dysregulation, such as wishful thinking, suppression, negative affect, and social insecurity (“Emotional-focused processes”), and (3) adaptive support-seeking coping strategies, including emotional support, instrumental support, and positive affect regulation (“social-focused processes”). This three-factor solution was used to operationalize cognitive-, emotional-, and social-focused candidate transdiagnostic process variables in all subsequent SEM analyses. Descriptive internal consistency estimates for the final indicator sets were acceptable, with Cronbach’s α = 0.75 for cognitive-focused processes, α = 0.70 for emotional-focused processes, and α = 0.76 for social-focused processes. Because these factors combined conceptually related but heterogeneous indicators, Cronbach’s α was interpreted descriptively and complemented by the CFA factor loadings.

Results of Confirmatory Factor Analysis suggesting 11 suitable predictors that loaded onto three higher-order cognitive-, emotional, and social-focused factors. Empathy, executive functioning, proactive coping, and difficulties maintaining friendships were excluded due to low and/or ambiguous factor loadings.

**Table 2:** Final model results from confirmatory factor analysis

Latent factor	Observed variable	λ	SE	*p*	*R*²
Cognitive-focused	Emotion regulation: cognitive reappraisal	0.442	0.033	< 0.001	0.195
Cognitive-focused	Active coping: perseverance	0.805	0.018	< 0.001	0.647
Cognitive-focused	Active coping: problem solving	0.668	0.018	< 0.001	0.447
Cognitive-focused	Coping flexibility	0.679	0.032	< 0.001	0.461
Emotional-focused	Uncertainty in social contact	0.604	0.027	< 0.001	0.365
Emotional-focused	Negative affect	0.675	0.028	< 0.001	0.455
Emotional-focused	Avoidance: repression	0.569	0.026	< 0.001	0.323
Emotional-focused	Avoidance: wishful thinking	0.560	0.027	< 0.001	0.313
Social-focused	Emotion regulation: expressive suppression	0.639	0.055	< 0.001	0.409
Social-focused	Instrumental support-seeking	0.897	0.053	< 0.001	0.804
Social-focused	Emotional support-seeking	0.785	0.065	< 0.001	0.617

Notes*.* λ = standardized factor loadings, SE = standard errors, *R*² = coefficient of determination

We used SEM to examine direct and indirect associations from various PTEs on mental health outcomes, while accounting for sociodemographic characteristics as covariates and perceived social support as a potential moderator. To address the second research objective, which was to examine the indirect association patterns involving the three identified higher-order factors (cognitive-, emotional-, and social-focused processes) in the associations between PTEs and mental health, we examined the indirect paths. We also explored differences in the occurrence and strength of associations among several types of PTEs (e.g., emotional abuse, physical neglect, and social exclusion), as outlined in research objective 2. To facilitate interpretation of the large number of indirect associations, Table 3 provides a summary matrix of statistically significant standardized indirect effects across PTE indicators, candidate transdiagnostic process domains, and mental health outcomes. Complete results for all indirect associations are provided in Supplementary Table S6.

### Direct effects

Among PTEs, social exclusion had the strongest direct association with several mental health outcomes, namely meaning of life (β = -0.33, *p* < .001), satisfaction with life (β = -0.34, *p* < .001), internalizing symptoms (β = 0.22, *p* < .001) and externalizing symptoms – substance use (β = -0.26, *p* = .004). Experiencing physical abuse was directly associated with lower levels of resilience (β = -0.21, *p* = .012). Other PTEs —including emotional neglect or abuse as well as sexual abuse, CLEs, discrimination and PTSD-related event—had no significant direct associations on the investigated mental health outcomes.

Among the process variables, both cognitive- and emotional-focused processes were directly associated with all six mental health outcomes with standardized estimates of β 0.20 to 0.37 for positive mental health and resilience (see Supplementary materials Table S4). The direct associations from emotional-focused processes to internalizing and externalizing symptoms revealed moderate to strong effects of β 0.35 to 0.61. Social-focused processes showed relatively weak direct associations, limited to positive mental health and internalizing symptoms (β 0.05 to 0.11).

### Moderation effects

Perceived social support attenuated negative associations between various PTEs and mental health outcomes. Specifically, perceived social support attenuated the negative effects of physical abuse on resilience (β = 0.18, *p* = .033) as well as on internalizing symptoms (β = -0.12, *p* = .039); from social exclusion on meaning of life (β = 0.25, *p* < .001) as well as satisfaction with life (β = 0.20, *p* = .004). However, perceived social support also strengthened the association between discrimination and externalizing personality (β = 0.17, *p* = .036). Further results on the moderating effects can be found in the Supplementary materials Table S5.

### Indirect effects

Numerous cross-sectional indirect associations were observed via cognitive-, emotional-, and social-focused processes, with emotional-focused processes showing the most consistent indirect association pattern across PTEs and mental health outcomes. Specifically, emotional-focused processes were relevant in indirect associations of childhood trauma (emotional neglect and abuse, sexual abuse), social CLEs, PTSD-related event, social exclusion, and discrimination with all of the five measured mental health outcomes (see Table 3). The strength of the indirect effect via emotional-focused processes in the three mentioned outcomes was particularly high in case of sexual abuse (SIE > 0.15) and social exclusion (SIE > 0.25), followed by discrimination, social CLEs, PTSD-related event and emotional abuse (SIE between 0.05 and 0.07). Noticeably, the highest indirect effects via emotional-focused processes appeared for the three psychopathological outcomes internalizing symptoms, externalizing personality and substance use.

Social CLEs, social exclusion, sexual abuse and emotional neglect were identified as PTEs that were indirectly linked to several mental health outcomes also via cognitive-, and social-focused processes. In general, the standardized indirect effect was relatively low for both cognitive- and social-focused processes, except for moderate values in case of social exclusion (SIE = 0.04) and internalizing as well as externalizing symptoms (personality, substance use). Notably, social-focused processes showed indirect associations primarily with positive mental health outcomes and internalizing symptoms.

Among the investigated PTEs, social exclusion showed the highest number of indirect effects (*n* = 15), followed by social CLEs and sexual abuse (both 8 indirect effects). These PTEs were partially mediated through all three psychological process factors. In contrast, associations involving emotional and physical abuse, PTSD-related event, and discrimination were primarily linked indirectly through by emotional-focused processes only. In contrast, physical neglect and health- and job-related critical life events showed no indirect associations with mental health outcomes, while physical abuse showed only one indirect association.

Overall, the three mediators most frequently contributed to indirect association patterns linking PTEs with mental health outcomes (satisfaction with life, meaning of life, and internalizing symptoms). In contrast, there were only few indirect associations between PTEs and externalizing factors (personality traits and substance use).

**Table 3:** Summary matrix of standardized indirect effects (SIEs) linking potentially traumatic event indicators with mental health outcomes through cognitive-, emotional-, and social-focused candidate transdiagnostic process variables (*N* = 3,051)

PTE indicator	Candidate transdiagnostic processes			Mental health outcomes					
	Cognitive-focused	Emotional-focused	Social-focused	Resilience	Meaning of life	Life satisfaction	Internalizing symptoms	Externalizing personality	Substance use
Emotional neglect	○	◔	○	○	○	○	○	○	○
Emotional abuse	○	◐	○	○	○	○	○	○	○
Sexual abuse	◔	●	○	○	○	○	●	●	◐
Discrimination	○	◐	○	○	◔	◔	◐	◐	◔
Social exclusion	◐	●	◔	◐	◐	●	●	●	●
PTSD-related event	○	◐	○	○	○	◔	◔	◔	○

Notes*.* White circles indicate non-significant associations after Benjamini–Hochberg correction. Increasingly darker shades represent larger standardized indirect effects, with dark grey indicating the strongest observed indirect associations. ○ = non-significant indirect effect (SIE), ◔ = small significant indirect effect 0.01 ≤ |SIE| < 0.10 ◐ = moderate significant indirect effect 0.10 ≤ |SIE| < 0.20, ● = large significant indirect effect |SIE| ≥ 0.20

## Discussion

This study examined direct and indirect associations between exposure to various potentially traumatic events (PTEs; e.g., emotional abuse, social exclusion) and mental health outcomes, including positive mental health and psychopathological symptoms, in a population-based sample of 3,051 emerging adults aged 16 to 25 years. Rather than testing causal mediation, the study examined whether theoretically informed transdiagnostic process variables were statistically positioned between PTE indicators and mental health outcomes, in line with established theoretical models and empirical evidence [24, 25, 33, 34]. Specifically, we aimed to (1) determine whether diverse psychological indicators cluster into higher-order transdiagnostic factors and (2) assess whether these factors account for shared variance between PTE indicators and mental health outcomes in indirect association models [21], (3) explore whether these associations differ by type of PTE, and (4) exploratorily examine whether perceived social support moderates direct associations between PTE indicators and mental health outcomes. We expected the results of this research to contribute to the expansion of the current state of research by considering various PTEs, mental health outcomes and psychological processes simultaneously, and to identify significant potential for prevention beyond disease-related boundaries.

Using 15 indicators derived from the HiTOP and RDoC frameworks, factor-analytic results supported a three-factor solution comprising 11 indicators, addressing the first research objective [23, 57]. These higher-order factors—cognitive-focused, emotional-focused, and social-focused processes—were moderately intercorrelated, indicating conceptual overlap while supporting their distinction (see Fig. 2). This structure is consistent with prior theoretical and empirical work identifying cognitive, emotional, and interpersonal processes as core transdiagnostic dimensions of psychopathology [24, 25, 33]. In this research, the cognitive-focused factor consists of cognitive reappraisal, two active coping strategies and coping flexibility. Two avoidant coping strategies, negative affective experience and uncertainty in social contact were summarized under an emotional-focused factor. The social-focused factor includes two support-seeking coping strategies and expressive suppression (see Fig. 2). Accordingly, the term transdiagnostic should be understood as referring to the framework-guided selection and cross-outcome relevance of these process variables, not as evidence that the identified factors represent definitive or clinically validated transdiagnostic mechanisms.

The theoretical distinction between cognitive-, emotional-, and social-focused processes should be interpreted as a heuristic organization of broad domains of functioning rather than as evidence of fully separable psychological systems. Several indicators considered in the present study could plausibly be assigned to more than one domain. For example, cognitive reappraisal is often conceptualized as a cognitive process whose primary function is emotion regulation, whereas expressive suppression has been described both as an emotion-regulation strategy and as a socially embedded process related to impression management and conformity to social norms [74, 75]. Similarly, support-seeking involves emotional, cognitive, and interpersonal components. The factor structure identified here therefore reflects a combination of theory-driven indicator selection and empirical clustering within the present sample. Future research should examine whether similar higher-order domains emerge across different populations, measures, and methodological approaches. Emotion regulation and cognitive reappraisal, representing the most frequently investigated mechanisms [24, 25, 33, 35, 72, 73], are central components of the emotional- and cognitive-focused factors in this research, respectively. While cognitive reappraisal loaded on the same factor together with rather cognitive-focused processes, previous conceptualizations have differed in their emphasis on whether the process is primarily cognitive or emotional. Consequently, it is often described as a cognitive process whose primary function is emotion regulation [74]. The same applies to expressive suppression, which was originally operationalized as emotion regulation but loaded onto the social-focused factor in this research. This is in line with the assumption that it is deeply intertwined with the social context and serves a social function such as managing social impressions or avoiding conflict [75]. However, expressive suppression has also been conceptualized as a response-focused emotion regulation strategy [74]. Nevertheless, factor labels should be regarded as provisional, and future research is needed to evaluate the robustness and generalizability of this structure across populations and measurement approaches.

Additionally, there are a few indications of heightened negative emotional reactivity (e.g., in response to stress) and uncertainty regarding emotional cues in social interaction that should be considered as transdiagnostic emotional processing [25, 33], closely related to the emotional-focused components of negative affective experience and uncertainty in social contact (i.e., heightened sensitivity to interpersonal rejection) in this research. This research also reflects the presumed explanatory role of further coping strategies for PTE with mental health-associations [25], as represented by active coping and coping flexibility (cognitive-focused processes), and support-seeking (social-focused processes).

Across all investigated PTEs, transdiagnostic process variables accounted for shared variance in the associations with mental health outcomes (research objective 2). Emotional-focused processes—characterized by maladaptive coping strategies and affective dysregulation—emerged as the most prominent candidate process variable, with small to moderate effect sizes. The emotional-focused factor was involved in indirect associations linking six out of eleven PTEs across all six mental health outcomes. The indirect association between sexual abuse and social exclusion (0.16 to 0.31) are particularly noteworthy compared to the otherwise rather small indirect effects. This pattern suggests that emotional-focused processes are strongly embedded in the cross-sectional association structure linking adversity indicators with both psychopathological symptoms and reduced well-being [35, 73]. Thus, emotional-focused processes should be examined further in future longitudinal and experimental research before conclusions can be drawn about their relevance for prevention and intervention [76].

Prior work suggests that difficulties in emotion regulation and reliance on maladaptive strategies are associated with increased risk for mental disorders [25]. Notably, dispositional avoidance—habitually evading emotionally challenging situations—has been strongly linked to depressive, anxiety, and eating disorders, especially when avoidance is not followed by engagement in problem-solving strategies [72, 77]. Supporting this, meta-analytic evidence points to moderate to high associations between avoidance and psychopathology [77]. Therapeutic approaches targeting emotion regulation, such as Emotion Regulation Group Therapy (ERGT) and Acceptance and Commitment Therapy (ACT), have demonstrated efficacy in reducing internalizing symptoms, borderline personality disorder, and substance use [72]. Similarly, Affect Regulation Training (ART) offers a promising transdiagnostic framework, although more evidence is needed [76]. School-based programs using CBT and mindfulness have shown positive mental health effects, particularly for at-risk adolescents, though outcomes vary in broader student populations [78], suggesting further potential for optimization in universal transdiagnostic promotion.

Although emotional-focused indirect associations predominated, indirect associations via cognitive- and social-focused processes were also observed [25]. These patterns appear particularly relevant for future research on interventions targeting resilience and the prevention of internalizing symptoms. Future studies may examine whether coping strategies across emotional, cognitive, and social domains prospectively predict changes in mental health after PTE exposure.

Overall, the magnitude of indirect associations varied across mental health outcomes: indirect effects were more pronounced for internalizing than externalizing symptoms, which were linked mainly by emotional-focused processes. This reflects the current literature’s emphasis on internalizing psychopathology and reveals a gap in understanding the mechanisms behind externalizing symptoms like substance use and impulsivity. Notably, emotional-focused processes also showed some of the strongest indirect associations with externalizing symptoms, particularly following socially contextualized PTEs such as exclusion and discrimination. This pattern suggests that emotional-focused processes should be further examined in longitudinal research on externalizing outcomes after adversity.

Beyond psychological factors, the role of neurobiological processes in externalizing outcomes remains underexplored. While trauma-exposed individuals often use substances as emotional self-medication, evidence also points to trauma-related neurobiological changes—such as disrupted dopamine systems—that affect emotion regulation and impulsivity [79]. Thus, alongside emotional processing, neurobiological mechanisms warrant further investigation in the development of externalizing symptoms after PTEs.

Influential pathways via cognitive and social-focused processes were less frequent and less pronounced than those via emotional-focused processes. However, these pathways appear particularly relevant in cases of PTEs in social contexts (i.e., critical life events, exclusion) and for positive mental health outcomes (i.e., satisfaction with life, meaning of life). Social and cognitive processes may be especially relevant for understanding PTEs that involve social contexts, because such experiences can shape how individuals perceive, interpret, and interact with their social environment, including their sense of belonging and perceived social support. However, the interpretation of these findings is difficult due to a lack of comparable study results.

Cognitive reappraisal, which involves reinterpreting a situation to alter its emotional impact and resembles to the cognitive-focused factor in this research, yielded mixed results in terms of its associations with psychopathology so far [77]. Nevertheless, it remains a core component of cognitive-behavioral therapy (CBT), with strong evidence supporting its effectiveness in treating internalizing disorders such as anxiety, somatoform, and eating disorders [80]. In contrast, expressive suppression, linked to the social-focused process in our findings, has been previously associated with moderate to strong relationships with internalizing symptoms [77].

Positive mental health and resilience remain comparatively understudied outcomes in transdiagnostic research [25, 81, 82]. While resilience is often assumed to arise from underlying mechanisms, most existing studies have focused on isolated factors [81]. The present findings support the need for more comprehensive approaches that extend beyond psychopathology by showing cross-sectional indirect associations between PTSD-related events and social exclusion with resilience via cognitive-focused processes such as cognitive flexibility. In line with prior research, the results point to psychological flexibility as a candidate process variable that may be relevant to resilience after adversity [83] According to Bonanno [83, 84], psychological flexibility requires a flexible mindset (motivation to confront adversity), context sensitivity (ability to select the most effective response given the situation), and a broad strategy repertoire (ability to adaptively shift strategies). Future research should explore how these elements function across cognitive, emotional, and social domains and how they contribute to positive mental health and resilience.

Addressing the third research objective, we identified meaningful differences in how distinct PTEs related to mental health outcomes. Indirect associations frequently exceeded direct effects, suggesting that the included psychological process variables captured substantial shared variance between PTE indicators and mental health outcomes. For instance, although emotional abuse and PTSD-related events showed no direct links to mental health outcomes, indirect associations via emotional-focused processes were evident. These patterns suggest that emotional-focused processes, such as detachment, maladaptive coping, and difficulties in social functioning, may be particularly relevant candidate processes in the association between interpersonal adversity and mental health. Rather than establishing these processes as causal mediators, the findings highlight their potential value for future longitudinal and experimental research and for the further development of screening and intervention approaches in high-risk groups. Conversely, physical abuse demonstrated a direct association with resilience, but transdiagnostic process variables contributed little to this association. This suggests that alternative candidate processes, potentially biological, contextual, or unmeasured psychosocial factors, should be explored in future research.

Social exclusion exhibited the strongest and most consistent associations across cognitive-, emotional-, and social-focused processes. In comparison to the overall relatively small indirect effects, the standardized values of the SIE for social exclusion via the emotional-focused factor were moderate, ranging from 0.26 to 0.31. These findings align with the PAST framework, which emphasizes threats to social belonging as core traumatic experiences [6]. The current results suggest that social-contextual adversities are strongly embedded in a broader network of cognitive-, emotional-, and social-focused process variables. However, longitudinal or experimental studies are needed to determine whether these processes represent causal mechanisms linking social exclusion to later mental health outcomes. Therefore, the DZPG Trialogical Board expanded the collaboratively developed MDS to include items prioritized by individuals with lived experience, such as stigma related to mental illness [51]. In line with the PAST framework, future research should examine whether stigma, similar to social exclusion and social critical life events, is associated with mental health outcomes through cognitive, emotional, and social transdiagnostic processes.

Perceived social support moderated several direct associations between PTE indicators and mental health outcomes. Consistent with previous research [24, 29], perceived social support attenuated associations of certain PTEs—such as physical abuse and social exclusion—with mental health. One counterintuitive finding warrants particular attention: higher perceived social support was associated with higher externalizing personality traits among individuals reporting discrimination. This pattern may point to more complex social dynamics in which some forms of social support do not necessarily have protective associations. For example, support embedded in peer contexts characterized by rule-breaking or maladaptive behavior may be associated with the normalization or reinforcement of externalizing tendencies, a process discussed in prior research on peer contagion and deviancy training [85]. Given the cross-sectional design, however, this interpretation remains speculative and should be examined in future longitudinal research.

Emerging adulthood represents a critical window for prevention, as vulnerability to trauma-related and internalizing symptoms is heightened during this developmental phase [15, 16]. In many areas, it is marked by a change in contact persons and medical care [15]. Despite an increased need for help, the use of support services for health in general and mental health problems in particular is rather low [86, 87]. Accordingly, public health strategies should prioritize early promotion of adaptive transdiagnostic skills and timely post-trauma support to prevent the consolidation of mental disorders.

### Clinical implications

From a clinical perspective, the findings identify emotional-focused processes as candidate transdiagnostic process variables that were prominently embedded in the cross-sectional association structure linking PTE indicators with mental health outcomes. Routine assessment of emotion regulation difficulties and maladaptive coping may therefore be clinically informative across diagnostic categories, particularly among young people with a history of social or interpersonal adversity. At the intervention level, the findings are consistent with the broader rationale for transdiagnostic and skills-based approaches that address emotion regulation capacities across multiple symptom domains. However, the present data do not establish emotional-focused processes as causal mechanisms or direct intervention targets. The strong associations observed for social exclusion further underscore the importance of assessing social stressors in clinical practice and considering social context alongside individual symptom profiles. Overall, these clinical implications should be regarded as hypothesis-generating and require confirmation in longitudinal and experimental studies.

### Limitations

Several limitations should be considered when interpreting these findings. First, the cross-sectional and retrospective design precludes causal inference. Although the models were theoretically specified with PTE indicators as predictors, transdiagnostic process variables as intervening variables, and mental health indicators as outcomes, the temporal and causal ordering of these constructs cannot be established. The observed indirect paths should therefore be interpreted as cross-sectional indirect associations rather than evidence of causal mediation. Longitudinal studies with repeated assessments are required to clarify the temporal ordering and causal mechanisms linking PTEs to psychopathology, mental well-being, and resilience [25].

Second, alternative associational interpretations cannot be ruled out [88]. The identified process variables may reflect consequences of current mental health symptoms rather than antecedent processes. They may also capture shared distress, negative affectivity, or self-report method variance. In addition, suppressor effects, collinearity among predictors and process variables, or reciprocal associations between PTEs, process variables, and mental health outcomes may have contributed to the observed patterns. Thus, the indirect paths should be understood as cross-sectional association structures, not as evidence that PTEs caused changes in process variables that subsequently caused mental health outcomes. It is also possible that these processes shape the likelihood of experiencing or reporting certain PTEs, particularly those involving social interactions. Future longitudinal and genetically informed studies are needed to clarify temporal ordering, disentangle shared liabilities and examine potential bidirectional pathways.

Third, several constructs in the present models are conceptually and empirically related. For example, negative affect, internalizing symptoms, and maladaptive coping may partly reflect shared distress or negative affectivity, and all variables were assessed by self-report. Shared method variance and construct overlap may therefore have contributed to the observed association patterns. Although the factor analytic approach helped organize the process indicators into empirically distinguishable higher-order factors, it cannot fully rule out redundancy between process variables and mental health outcomes.

Fourth, the absence of a gold standard for assessing transdiagnostic mechanisms limits measurement precision. Future research should prioritize the development and validation of instruments capable of reliably capturing higher-order transdiagnostic factors across domains. The MDS initiative within the DZPG, along with higher-order factors identified here, represents a first important step toward this goal. The factor solution is conditional on the selected item set and modeling decisions. Although residual covariances and indicator exclusions improved model fit and factorial clarity, these decisions may also affect construct validity and the comparability of the factors with other operationalizations of transdiagnostic processes.

Fifth, this study—like much prior transdiagnostic research—focused primarily on individual-level mechanisms, despite their embeddedness in broader social and societal contexts [81]. Interactions with ecological, structural, and societal factors (e.g., access to resources, institutional support, or systemic inequalities) were not examined and warrant greater attention in future research adopting a multisystemic resilience perspective [82]. Furthermore, all constructs were assessed using self-report measures, which may be subject to recall and reporting biases. Future studies would benefit from integrating self-reports with behavioral tasks, cognitive assessments, and biological indicators (e.g., neuroendocrine or inflammatory markers). Such multi-method approaches would provide a more comprehensive understanding of the association patterns linking PTEs to mental health and help identify domains with the greatest unexplained variance.

Fifth, the observed associations may partly reflect pre-existing individual differences that were not measured in the present study. Differences in transdiagnostic process variables may have been present before the occurrence of the reported PTEs, and shared genetic, temperamental, familial, or early environmental influences may contribute both to exposure to certain PTEs and to subsequent mental health outcomes. Consequently, the identified indirect association patterns may partly reflect common underlying liabilities rather than processes arising as a consequence of adversity exposure. Future genetically informed, longitudinal, and family-based designs are needed to disentangle these possibilities.

## Conclusions

Understanding shared transdiagnostic process variables—such as emotion dysregulation and maladaptive coping—may provide insight into the substantial overlap observed across mental health outcomes. In this study, emotional-focused processes emerged as the most prominent candidate process variables in the cross-sectional association structure linking PTE indicators with mental health outcomes.

Socially contextualized adversities, particularly social exclusion, showed especially consistent direct and indirect associations with psychopathology, positive mental health, and resilience. By incorporating positive mental health and resilience outcomes, the findings broaden the scope of transdiagnostic research beyond symptom-focused outcomes and generate hypotheses for future longitudinal and experimental studies.

Accordingly, the present findings may help prioritize emotional-, cognitive-, and social-focused processes for future research on trauma-related mental health. Conclusions about prevention, intervention, or clinical targeting require designs that can establish temporal ordering and test whether modifying these processes changes subsequent mental health outcomes. Reliable assessment tools, longitudinal data, and intervention studies are needed before these candidate processes can be translated into routine public health or clinical practice.

## Supplementary Information

Below is the link to the electronic supplementary material.

Supplementary Material 1

## Data Availability

The datasets generated and analyzed during the current study are available as a scientific use file for non-commercial use upon request from the Research Data Centre at the Robert Koch Institute. Requests should be directed to [fdz@rki.de](mailto: fdz@rki.de) . The underlying code for this study is provided in the Supplementary materials file S7.

## **Abbreviations**

ACT: Acceptance and Commitment Therapy
ART: Affect Regulation Training
BH: Benjamini–Hochberg
CBT: Cognitive-behavioral therapy
CFA: Confirmatory factor analysis
CFI: Comparative Fit Index
DZPG: Deutsches Zentrum für Psychische Gesundheit (German Centre for Mental Health)
ERGT: Emotion Regulation Group Therapy
EFA: Exploratory factor analysis
FDR: False discovery rate
HiTOP: Hierarchical Taxonomy of Psychopathology
JEPSY: Studie zur psychischen Gesundheit von jungen Erwachsenen in Deutschland (Study on the Mental Health of Young Adults in Germany)
KMO: Kaiser-Meyer-Olkin criterion
KiGGS: Studie zur Gesundheit von Kindern und Jugendlichen in Deutschland (German Health Interview and Examination Survey for Children and Adolescents)
MDS: Minimal Data Set
OSS: Oslo Social Support Scale
PAST: Physical and Social Trauma framework
PTEs: Potentially traumatic events
PTSD: Post-traumatic stress disorder
RDoC: Research Domain Criteria
RMSEA: Root Mean Square Error of Approximation
MLR: Maximum likelihood estimation
SRMR: Standardized Root Mean Square Residual
SEM: Structural equation model
SD: Standard deviation
SIE: Standardized Indirect Effect
TLI: Tucker-Lewis Index
