# Supplementary material for: Candidate transdiagnostic processes linking potentially traumatic experiences to psychopathology, mental well-being and resilience in emerging adults
Source: BMC Psychiatry. 2026 Jun 22;26:484. doi: 10.1186/s12888-026-08302-8 (PMC13289464; doi:10.1186/s12888-026-08302-8)
Supplement: Supplementary file 1 — Supplementary Material 1 [file 12888_2026_8302_MOESM1_ESM.docx]

**Supplementary Information.**

# Candidate transdiagnostic processes linking potentially traumatic experiences to psychopathology, mental well-being and resilience in emerging adults

**Table S1. Overview of outcome and potentially traumatic event measures, item sources, response formats, and scoring**

| **Analytic construct** | **Variable label (R code)** | **Full instrument name** | **Abbreviation** | **Complete scale or selected items** | **No. of items used** | **Item content/domain** | **Response format** | **Coding / scoring** | **Used as** |
| --- | --- | --- | --- | --- | --- | --- | --- | --- | --- |
| Resilience | J_LGcdrisc_Score | Connor-Davidson Resilience Scale, 2-item version | CD-RISC-2 | Complete short scale | 2 | Resilience/adaptability after adversity; recovery after stress or hardship. | Likert-type response scale | Mean score; higher values indicate higher resilience | Outcome |
| Life satisfaction | J_LQzufrC10 | General life satisfaction item |  | Single item | 1 | Overall life satisfaction | 0 = not at all to 10 = completely | Higher values indicate higher life satisfaction | Outcome |
| Meaning in life | J_LQzufr17 | Meaning in Life Questionnaire, presence item | MLQ | Selected item | 1 | Perceived meaning in life | 0 = not at all to 10 = completely | Higher values indicate greater meaning in life | Outcome |
| Internalizing symptoms | J_ScoreInt | Patient Health Questionnaire-4 and DSM-5-TR cross-cutting symptom items | PHQ-4; DSM-5-TR | Selected items | 10 | Anxiety and depressive symptoms, including nervousness and anhedonia | Instrument-specific response format | Factor score, min-max standardized to 0–1; higher values indicate greater symptom severity | Outcome |
| Externalizing symptoms: personality traits | J_ScoreExt | Personality Inventory for DSM-5 and DSM-5-TR items | PID-5; DSM-5-TR | Selected items | 5 | Disinhibition-related traits, including risk-taking and impulsivity | Instrument-specific response format | Factor score, min-max standardized to 0–1; higher values indicate greater symptom severity | Outcome |
| Externalizing symptoms: substance use | J_ScoreSub | DSM-5-TR cross-cutting symptom items | DSM-5-TR | Selected items | 4 | Alcohol, tobacco, and non-prescription drug use | Instrument-specific response format | Factor score, min-max standardized to 0–1; higher values indicate greater symptom severity | Outcome |
|  |  |  |  |  |  |  |  |  |  |
| Emotional neglect | J_SFcts1_kat | Childhood Trauma Screener | CTS | Complete screener, item-level indicators used | 1 of 5 | Emotional neglect | 0 = never to 4 = very often | Dichotomized indicator; 1 = exposure present | Predictor |
| Emotional abuse | J_SFcts2_kat | Childhood Trauma Screener | CTS | Complete screener, item-level indicators used | 1 of 5 | Emotional abuse | 0 = never to 4 = very often | Dichotomized indicator; 1 = exposure present | Predictor |
| Physical neglect | J_SFcts3_kat | Childhood Trauma Screener | CTS | Complete screener, item-level indicators used | 1 of 5 | Physical neglect | 0 = never to 4 = very often | Dichotomized indicator; 1 = exposure present | Predictor |
| Physical abuse | J_SFcts4_kat | Childhood Trauma Screener | CTS | Complete screener, item-level indicators used | 1 of 5 | Physical abuse | 0 = never to 4 = very often | Dichotomized indicator; 1 = exposure present | Predictor |
| Sexual abuse | J_SFcts5_kat | Childhood Trauma Screener | CTS | Complete screener, item-level indicators used | 1 of 5 | Sexual abuse | 0 = never to 4 = very often | Dichotomized indicator; 1 = exposure present | Predictor |
| PTSD-related event | J_PKptbs | Primary Care PTSD Screen for DSM-5 | PC-PTSD-5 | Complete screener | 5 | Lifetime exposure to frightening, horrible, or traumatic event and PTSD-related symptoms | yes/no | Cut-off of 3 out of 5 symptoms; 1 = PTSD-related event | Predictor |
| Social critical life event | J_BLevent_Soz | Social Readjustment Rating Scale-based critical life event items | SRRS/CLE | Selected items | 4 | Social-domain critical life events: separation/ divorce, marriage, leaving home, having own child/ adopt | yes/no or count; please verify | 1 = at least one social CLE in past 12 months | Predictor |
| Occupational critical life event | J_BLevent_Ber | Social Readjustment Rating Scale-based critical life event items | SRRS/CLE | Selected items | 2 | Occupational-domain critical life events: professional qualification, job loss | yes/no or count; please verify | 1 = at least one occupational CLE in past 12 months | Predictor |
| Health-related critical life event | J_BLevent_Ges | Social Readjustment Rating Scale-based critical life event items | SRRS/CLE | Selected items | 3 | Health-domain critical life events: death, severe illness or accident | yes/no or count; please verify | 1 = at least one health-related CLE in past 12 months | Predictor |
| Discrimination | J_SFdis | Minimum Data Set item developed by Tschorn et al. | MDS | Single item | 1 | Perceived discrimination during the past 30 days | Visual analogue scale, 0–100 | Higher values indicate more perceived discrimination; normalized to 0–1 for standardized indirect effects | Predictor |
| Social exclusion | J_SFexklu | Minimum Data Set item developed by Tschorn et al. | MDS | Single item | 1 | Perceived social exclusion during the past 30 days | Visual analogue scale, 0–100 | Higher values indicate more perceived social exclusion; normalized to 0–1 for standardized indirect effects | Predictor |

**Table S2. Overview of transdiagnostic process indicators, source instruments, coding direction, and factor assignment**

| **Higher-order factor** | **Variable label (R code)** | **Full instrument name** | **Abbreviation** | **Complete scale or selected items** | **No. of items used** | **Item content/domain** | **Coding direction in analyses** | **Included in final CFA/SEM** |
| --- | --- | --- | --- | --- | --- | --- | --- | --- |
| Cognitive-focused processes | J_SFerq_ScoreNB | Emotion Regulation Questionnaire, cognitive reappraisal subscale | ERQ | Complete subscale or selected subscale score | 4 | Cognitive reappraisal | Higher values = more adaptive functioning | Yes |
| Cognitive-focused processes | J_COPE_PER | Short Adult Coping Scale, active coping: perseverance | SACS-16 | Selected two-item subscale | 2 | Perseverance/active coping | Higher values = more adaptive functioning | Yes |
| Cognitive-focused processes | J_COPE_PRO | Short Adult Coping Scale, active coping: problem solving | SACS-16 | Selected two-item subscale | 2 | Problem-solving/active coping | Higher values = more adaptive functioning | Yes |
| Cognitive-focused processes | J_COPE_FLE | Short Adult Coping Scale, coping flexibility | SACS-16 | Selected two-item subscale | 2 | Flexible use of coping strategies | Higher values = more adaptive functioning | Yes |
| Emotional-focused processes | J_SFipsm_Score_rev | Interpersonal Sensitivity Measure, uncertainty in social contact | IPSM | Selected items | 4 | Social uncertainty/insecurity in interpersonal contact | Reverse-coded; higher values = lower uncertainty / more adaptive functioning | Yes |
| Emotional-focused processes | J_SFpanas_Neg_rev | Positive and Negative Affect Schedule, negative affect | PANAS | Selected items | 2 | Negative affect | Reverse-coded; higher values = lower negative affect / more adaptive functioning | Yes |
| Emotional-focused processes | J_COPE_REP_rev | Short Adult Coping Scale, avoidance: repression | SACS-16 | Selected two-item subscale | 2 | Repressive/avoidant coping | Reverse-coded; higher values = less repression / more adaptive functioning | Yes |
| Emotional-focused processes | J_COPE_WIS_rev | Short Adult Coping Scale, avoidance: wishful thinking | SACS-16 | Selected two-item subscale | 2 | Wishful-thinking coping | Reverse-coded; higher values = less wishful thinking / more adaptive functioning | Yes |
| Social-focused processes | J_SFerq_ScoreUD_rev | Emotion Regulation Questionnaire, expressive suppression subscale | ERQ | Complete subscale or selected subscale score | 4 | Expressive suppression | Reverse-coded; higher values = lower suppression / more adaptive functioning | Yes |
| Social-focused processes | J_COPE_INS | Short Adult Coping Scale, instrumental support seeking | SACS-16 | Selected two-item subscale | 2 | Seeking instrumental support | Higher values = more adaptive functioning | Yes |
| Social-focused processes | J_COPE_EMO | Short Adult Coping Scale, emotional support seeking | SACS-16 | Selected two-item subscale | 2 | Seeking emotional support | Higher values = more adaptive functioning | Yes |
| Excluded after EFA/CFA | insert variable | Level of Personality Functioning Scale-Brief Form | LPFS-BF | Selected item | 1 | Empathy | Higher values = more adaptive functioning | Excluded due to weak/ambiguous loading |
| Excluded after EFA/CFA | insert variable | Behavior Rating Inventory of Executive Function-Adult Version / DSM-5-TR item set | BRIEF-A / DSM-5-TR | Selected items | 3 | Executive functioning | Higher values = more adaptive functioning | Excluded due to weak/ambiguous loading |
| Excluded after EFA/CFA | J_COPE_PROACTIVE | Short Adult Coping Scale, proactive coping | SACS-16 | Selected two-item subscale | 2 | Proactive coping | Higher values = more adaptive functioning | Excluded in CFA |
| Excluded after EFA/CFA | insert variable | WHO Disability Assessment Schedule 2.0 | WHO-DAS 2.0 | Selected item | 1 | Ability to maintain friendships | Higher values = more adaptive functioning | Excluded in CFA |

**S3. Results from testing of requirements for performing Structural Equation Modeling**

Before fitting the structural equation models, we systematically evaluated the core statistical assumptions—multicollinearity, normality of residuals, and homoscedasticity—using the full sample (N = 3,051).

Collinearity diagnostics were based on variance-inflation factors (VIFs) obtained from a multiple regression that included all manifest predictors and interaction terms to be entered in the SEM. All VIFs ranged from 1.07 to 2.09, below the conventional threshold of 10 (and even the more stringent cut-off of 5). We therefore concluded that multicollinearity is negligible and unlikely to bias parameter estimates.

Univariate normality of model residuals was assessed with the Shapiro–Wilk test (W = 0.993, p < .001). The Q–Q plot indicated an approximately normal distribution of residuals, with minor deviations at the tails (Figure S1.1). Given that even trivial deviations reach significance in large samples, we adopted a robust maximum-likelihood estimator with Huber–White (sandwich) standard errors and Satorra–Bentler corrections (MLR) for all SEMs. This estimator provides unbiased point estimates under non-normality and yields standard errors and fit statistics that are robust to both non-normality and heteroscedasticity.

The studentized Breusch–Pagan test indicated significant heteroscedasticity (BP = 125.98, df = 24, p < .001). The residuals vs. fitted values plot showed no substantial heteroscedasticity or systematic pattern, suggesting homogeneity of variance (Figure S1.2). A slight increase in the spread of residuals at lower fitted values was observed, but overall, the model assumptions were deemed sufficiently met. Because robust (MLR) estimation corrects standard errors for heteroscedasticity, no further transformation was deemed necessary. For additional assurance, key indirect effects were replicated with non-parametric percentile-bootstrap confidence intervals (5,000 draws); the pattern of significance did not change.


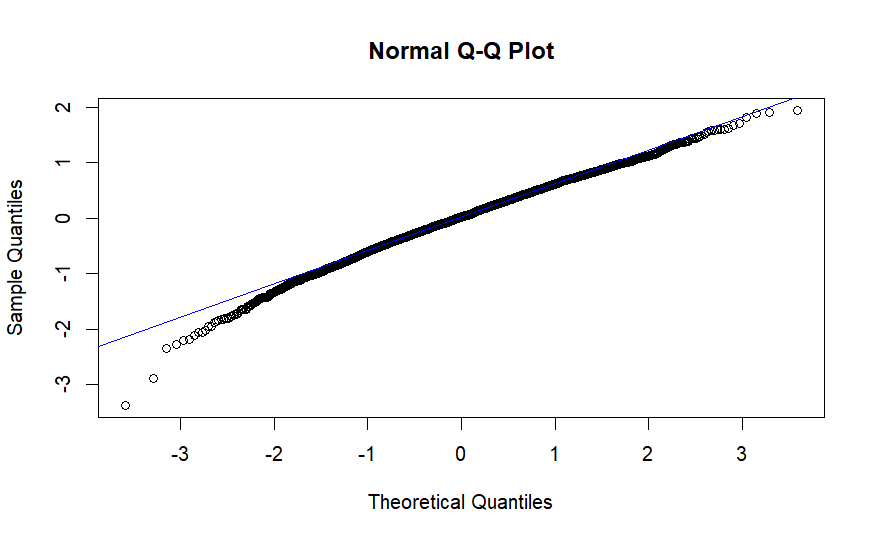


Figure S3.1. *Normal Q–Q plot of model residuals suggesting approximately normal distribution*


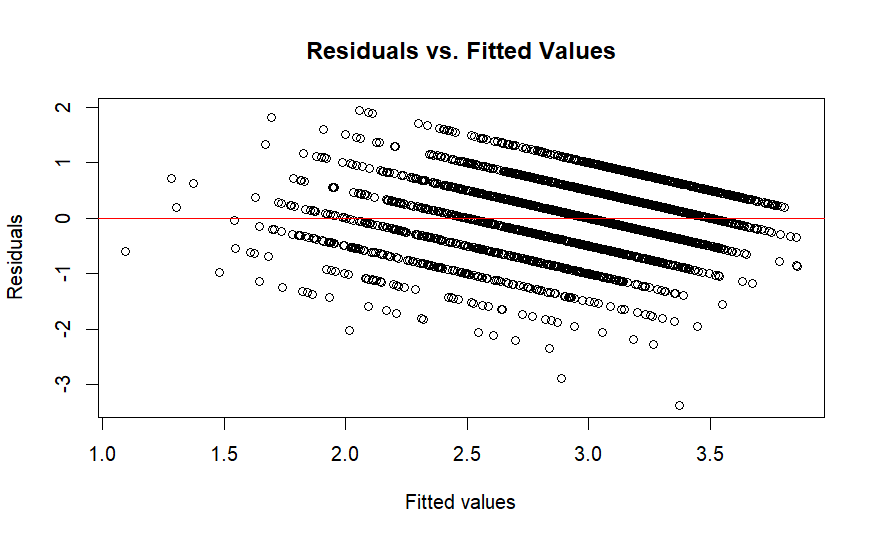


Figure S3.2. *Residuals vs. fitted values plot suggesting homogeneity of variance*

Taken together, diagnostics suggest that (a) multicollinearity is negligible, (b) deviations from normality are minor and accounted for by robust estimation, and (c) heteroscedasticity is sufficiently addressed through the use of MLR. Accordingly, the application of SEM appears appropriate given the distributional properties of the data and the corrective measures employed.

**Table S4. Descriptive statistics for mental health outcomes, indications for experience of potentially traumatic events and the final selection of mediators (emotional, social, cognitive processes) resulting from factorial analyses (N=3,051)**

|  | Weighted | | |  | | Unweighted | | | | | | |
| --- | --- | --- | --- | --- | --- | --- | --- | --- | --- | --- | --- | --- |
| Variable | Mean | Prop. | 95% CI | |  | | Mean | Prop. | | | 95% CI |  |
| Resilience | 2.90 |  | [2.85 – 2.95] | |  | 2.95 | | |  | [2.92 – 2.97] | | |
| Meaning of life | 6.52 |  | [6.37 – 6.66] | |  | 6.73 | | |  | [6.65 – 6.81] | | |
| Life satisfaction | 6.57 |  | [6.43 – 6.71] | |  | 6.72 | | |  | [6.65 – 6.79] | | |
| Internalizing symptoms | 0.29 |  | [0.28 – 0.30] | |  | 0.29 | | |  | [0.28 – 0.30] | | |
| Externalizing personality | 0.24 |  | [0.23 – 0.25] | |  | 0.23 | | |  | [0.23 – 0.24] | | |
| Substance use | 0.14 |  | [0.13 – 0.15] | |  | 0.12 | | |  | [0.12 – 0.13] | | |
| Occupational CLE | 0.18 |  | [0.16 – 0.21] | |  | 0.15 | | |  | [0.14 – 0.17] | | |
| Health-related CLE | 0.34 |  | [0.31 – 0.38] | |  | 0.35 | | |  | [0.33 – 0.38] | | |
| Social CLE | 0.27 |  | [0.24 – 0.31] | |  | 0.28 | | |  | [0.26 – 0.29] | | |
| Emotional neglect |  | 7.39 | [0.07 – 0.07] | |  |  | | | 5.77 | [0.05 – 0.07] | | |
| Emotional abuse |  | 22.18 | [0.22 – 0.22] | |  |  | | | 19.35 | [0.18 – 0.21] | | |
| Physical neglect |  | 12.34 | [0.12 – 0.12] | |  |  | | | 9.61 | [0.09 – 0.11] | | |
| Physical abuse |  | 6.06 | [0.06 – 0.06] | |  |  | | | 4.36 | [0.04 – 0.05] | | |
| Sexual abuse |  | 8.54 | [0.09 – 0.09] | |  |  | | | 10.53 | [0.09 – 0.12] | | |
| PTSD-related events |  | 24.52 | [0.24 – 0.25] | |  |  | | | 23.05 | [0.22 – 0.25] | | |
| Discrimination | 9.66 |  | [8.43 – 10.90] | |  | 9.14 | | |  | [8.53 – 9.76] | | |
| Social exclusion | 29.08 |  | [27.35 – 30.82] | |  | 28.62 | | |  | [27.65 – 29.59] | | |
| Social Support | 9.40 |  | [9.27 – 9.53] | |  | 9.54 | | |  | [9.47 – 9.62] | | |
| Cognitive-focused | 6.13 |  | [6.03 – 6.23] | |  | 6.22 | | |  | [6.17 – 6.28] | | |
| Emotion-focused | 2.19 |  | [2.16 – 2.23] | |  | 2.20 | | |  | [2.18 – 2.22] | | |
| Social-focused | 7.33 |  | [7.20 – 7.46] | |  | 7.57 | | |  | [7.50 – 7.65] | | |

*Notes*. Prop. = proportion %, CLE = critical life event

**Table S5. Direct, moderated and total effects from potentially traumatic events (N=3,051) on resilience, meaning of life, satisfaction with life, internalizing symptoms, externalizing symptoms (personality traits, substance use)**

| **Predictor** | | | ***B*** | | | ***SE*** | **95% *CI*** | | | | **β** | ***p*** | ***p (BH)*** | |  |
| --- | --- | --- | --- | --- | --- | --- | --- | --- | --- | --- | --- | --- | --- | --- | --- |
| **Resilience** | | | | | | | | | | | | |  | |  |
| **Direct effects** |  | | | |  | |  | | | |  |  |  | |  |
| Cognitive-focused | **0.10** | | | | 0.01 | | 0.09 - 0.12 | | | | 0.33 | <0.001 | <0.001 | |  |
| Emotional-focused | **0.32** | | | | 0.05 | | 0.22 - 0.42 | | | | 0.20 | <0.001 | <0.001 | |  |
| Social-focused | 0.00 | | | | 0.01 | | -0.01 - 0.01 | | | | 0.01 | 0.823 | 0.968 | |  |
| Occupational CLE^1^ | -0.04 | | | | 0.17 | | -0.37 - 0.28 | | | | -0.02 | 0.781 | 0.968 | |  |
| Health-related CLE^1^ | -0.17 | | | | 0.10 | | -0.36 - 0.03 | | | | -0.14 | 0.092 | 0.302 | |  |
| Social CLE^1^ | -0.02 | | | | 0.12 | | -0.25 - 0.21 | | | | -0.01 | 0.880 | 0.978 | |  |
| Emotional neglect^1^ | -0.01 | | | | 0.25 | | -0.50 - 0.49 | | | | <-0.01 | 0.959 | 0.983 | |  |
| Emotional abuse^1^ | 0.23 | | | | 0.17 | | -0.11 - 0.55 | | | | 0.12 | 0.171 | 0.391 | |  |
| Physical neglect^1^ | -0.20 | | | | 0.24 | | -0.67 - 0.25 | | | | -0.08 | 0.415 | 0.638 | |  |
| Physical abuse^1^ | **-0.76** | | | | 0.30 | | -1.34 - -0.18 | | | | -0.21 | 0.013 | 0.064 | |  |
| Sexual abuse^1^ | -0.11 | | | | 0.20 | | -0.49 - 0.28 | | | | -0.05 | 0.569 | 0.813 | |  |
| Discrimination | 0.00 | | | | <0.01 | | -0.01 - 0.01 | | | | 0.02 | 0.818 | 0.968 | |  |
| Social exclusion | 0.00 | | | | <0.01 | | -0.01 - 0.00 | | | | -0.13 | 0.106 | 0.302 | |  |
| PTSD-related event^1^ | -0.18 | | | | 0.15 | | -0.48 - 0.10 | | | | -0.10 | 0.196 | 0.391 | |  |
| Age | -0.01 | | | | 0.01 | | -0.02 - 0.01 | | | | -0.02 | 0.283 | 0.514 | |  |
| Sex (ref. male) | 0.00 | | | | 0.03 | | -0.06 - 0.06 | | | | 0.00 | 0.983 | 0.514 | |  |
| Medium education (ref. low) | 0.15 | | | | 0.12 | | -0.06 - 0.40 | | | | 0.08 | 0.176 | 0.391 | |  |
| High education (ref. low) | 0.18 | | | | 0.11 | | -0.03 - 0.42 | | | | 0.11 | 0.095 | 0.302 | |  |
| In Education (ref. low) | 0.12 | | | | 0.12 | | -0.11 - 0.36 | | | | 0.05 | 0.321 | 0.535 | |  |
| **Moderation effects** |  | | | |  | |  | | | |  |  |  | |  |
| Occupational CLE x SOC | 0.01 | | | | 0.02 | | -0.02 - 0.04 | | | | 0.05 | 0.529 | 0.765 | |  |
| Health-related CLE x SOC | 0.02 | | | | 0.01 | | 0.00 - 0.04 | | | | 0.13 | 0.097 | 0.342 | |  |
| Social CLE x SOC | 0.00 | | | | 0.01 | | -0.02 - 0.03 | | | | 0.01 | 0.899 | 0..899 | |  |
| Emotional neglect x SOC | -0.01 | | | | 0.03 | | -0.07 - 0.05 | | | | -0.03 | 0.743 | 0.8818 | |  |
| Emotional abuse x SOC | -0.03 | | | | 0.02 | | -0.06 - 0.01 | | | | -0.13 | 0.143 | 0.342 | |  |
| Physical neglect x SOC | 0.02 | | | | 0.03 | | -0.03 - 0.07 | | | | 0.08 | 0.396 | 0.765 | |  |
| Physical abuse x SOC | **0.08** | | | | 0.04 | | 0.01 - 0.14 | | | | 0.18 | 0.032 | 0.342 | |  |
| Sexual abuse x SOC | 0.01 | | | | 0.02 | | -0.03 - 0.05 | | | | 0.03 | 0.686 | 0.818 | |  |
| Discrimination x SOC | 0.00 | | | | 0.00 | | 0.00 - 0.00 | | | | -0.05 | 0.556 | 0.765 | |  |
| Social exclusion x SOC | 0.00 | | | | 0.00 | | 0.00 - 0.00 | | | | 0.10 | 0.155 | 0.342 | |  |
| PTSD-related event x SOC | 0.03 | | | | 0.01 | | 0.00 - 0.06 | | | | 0.14 | 0.063 | 0.342 | |  |
| **Total effects** |  | | | |  | |  | | | |  |  |  | |  |
| Occupational CLE^1^ | -0.03 | | | | 0.17 | | -0.36 - 0.31 | | | | -0.01 | 0.862 |  | |  |
| Health-related CLE^1^ | -0.16 | | | | 0.10 | | -0.36 - 0.03 | | | | -0.13 | 0.101 |  | |  |
| Social CLE^1^ | 0.00 | | | | 0.12 | | -0.24 - 0.23 | | | | <-0.01 | 0.976 |  | |  |
| Emotional neglect^1^ | -0.06 | | | | 0.26 | | -0.55 - 0.44 | | | | -0.02 | 0.812 |  | |  |
| Emotional abuse^1^ | -0.13 | | | | 0.20 | | -0.53 - 0.26 | | | | -0.06 | 0.497 |  | |  |
| Physical neglect^1^ | **-0.76** | | | | 0.31 | | -1.35 - -0.15 | | | | -0.21 | 0.014 |  | |  |
| Physical abuse^1^ | -0.20 | | | | 0.23 | | -0.67 - 0.24 | | | | -0.08 | 0.396 |  | |  |
| Sexual abuse^1^ | 0.15 | | | | 0.17 | | -0.19 - 0.46 | | | | 0.08 | 0.383 |  | |  |
| Discrimination | 0.00 | | | | <0.01 | | -0.01 - 0.01 | | | | -0.02 | 0.838 |  | |  |
| Social exclusion | **-0.01** | | | | <0.01 | | -0.01 - 0.00 | | | | -0.30 | <0.001 |  | |  |
| PTSD-related event^1^ | -0.21 | | | | 0.15 | | -0.51 - 0.06 | | | | -0.12 | 0.136 |  | |  |
|  |  | | | |  | |  | | | |  |  |  | |  |
| **Predictor** | ***B*** | | | | ***SE*** | | **95% *CI*** | | | | **β** | ***p*** | ***p (BH)*** | |  |
| **Meaning of Life** | | | | | | | | | | | | |  | |  |
| **Direct effects** |  | | | |  | |  | | | |  |  |  | |  |
| Cognitive-focused | **0.30** | | | | 0.03 | | 0.25 - 0.35 | | | | 0.32 | <0.001 | <0.001 | |  |
| Emotional-focused | **1.22** | | | | 0.15 | | 0.95 - 1.53 | | | | 0.26 | <0.001 | <0.001 | |  |
| Social-focused | **0.07** | | | | 0.02 | | 0.04 - 0.10 | | | | 0.11 | <0.001 | <0.001 | |  |
| Occupational CLE^1^ | -0.23 | | | | 0.51 | | -1.23 - 0.83 | | | | -0.04 | 0.648 | 0.926 | |  |
| Health-related CLE^1^ | -0.13 | | | | 0.26 | | -0.64 - 0.40 | | | | -0.04 | 0.633 | 0.926 | |  |
| Social CLE^1^ | -0.01 | | | | 0.34 | | -0.66 - 0.65 | | | | <-0.01 | 0.974 | 0.984 | |  |
| Emotional neglect^1^ | -0.17 | | | | 0.91 | | -1.96 - 1.70 | | | | -0.02 | 0.855 | 0.977 | |  |
| Emotional abuse^1^ | -0.13 | | | | 0.51 | | -1.13 - 0.85 | | | | -0.02 | 0.794 | 0.977 | |  |
| Physical neglect^1^ | -0.34 | | | | 0.56 | | -1.42 - 0.80 | | | | -0.05 | 0.544 | 0.870 | |  |
| Physical abuse^1^ | -0.02 | | | | 1.00 | | -2.01 - 1.88 | | | | <-0.01 | 0.984 | 0.984 | |  |
| Sexual abuse^1^ | 0.24 | | | | 0.62 | | -1.01 - 1.46 | | | | 0.03 | 0.701 | 0.967 | |  |
| Discrimination | 0.01 | | | | 0.01 | | -0.01 - 0.03 | | | | 0.08 | 0.299 | 0.630 | |  |
| Social exclusion | **-0.03** | | | | 0.01 | | -0.04 - -0.01 | | | | -0.33 | <0.001 | <0.001 | |  |
| PTSD-related event^1^ | -0.30 | | | | 0.44 | | -1.15 - 0.55 | | | | -0.06 | 0.527 | 0.870 | |  |
| Age | **-0.03** | | | | 0.02 | | -0.06 - -0.01 | | | | -0.04 | 0.020 | 0.071 | |  |
| Sex (ref. male) | **0.42** | | | | 0.08 | | 0.27 - 0.58 | | | | 0.09 | <0.001 | <0.001 | |  |
| Medium education (ref. low) | 0.22 | | | | 0.30 | | -0.40 - 0.81 | | | | 0.04 | 0.480 | 0.835 | |  |
| High edocation (ref. low) | -0.06 | | | | 0.30 | | -0.65 - 0.54 | | | | -0.01 | 0.849 | 0.977 | |  |
| In Education (ref. low) | -0.27 | | | | 0.32 | | -0.93 - 0.33 | | | | -0.04 | 0.397 | 0.754 | |  |
| **Moderation effects** |  | | | |  | |  | | | |  |  |  | |  |
| Occupational x SOC | 0.04 | | | | 0.05 | | -0.07 - 0.14 | | | | 0.06 | 0.459 | 0.918 | |  |
| Health-related x SOC | 0.02 | | | | 0.03 | | -0.03 - 0.07 | | | | 0.06 | 0.449 | 0.918 | |  |
| Social x SOC | 0.01 | | | | 0.03 | | -0.06 - 0.07 | | | | 0.01 | 0.856 | 0.946 | |  |
| Emotional neglect x SOC | -0.01 | | | | 0.11 | | -0.24 - 0.21 | | | | -0.01 | 0.903 | 0.946 | |  |
| Emotional abuse x SOC | -0.02 | | | | 0.06 | | -0.13 - 0.09 | | | | -0.04 | 0.680 | 0.940 | |  |
| Physical neglect x SOC | 0.03 | | | | 0.06 | | -0.09 - 0.15 | | | | 0.04 | 0.606 | 0.940 | |  |
| Physical abuse x SOC | 0.03 | | | | 0.11 | | -0.18 - 0.25 | | | | 0.03 | 0.769 | 0.940 | |  |
| Sexual abuse x SOC | 0.00 | | | | 0.07 | | -0.13 - 0.13 | | | | <-0.01 | 0.963 | 0.963 | |  |
| Discrimination x SOC | 0.00 | | | | <0.01 | | 0.00 - 0.00 | | | | -0.07 | 0.376 | 0.918 | |  |
| Social exclusion x SOC | **0.00** | | | | <0.01 | | 0.00 - 0.00 | | | | 0.25 | <0.001 | 0.010 | |  |
| PTSD-related event x SOC | 0.04 | | | | 0.05 | | -0.05 - 0.13 | | | | 0.08 | 0.362 | 0.918 | |  |
| **Total effects** |  | | | |  | |  | | | |  |  |  | |  |
| Social CLE^1^ | 0.04 | | | | 0.34 | | -0.60 - 0.72 | | | | 0.01 | 0.901 |  | |  |
| Occupational CLE^1^ | -0.16 | | | | 0.52 | | -1.16 - 0.89 | | | | -0.03 | 0.749 |  | |  |
| Health CLE^1^ | -0.12 | | | | 0.26 | | -0.65 - 0.43 | | | | -0.03 | 0.646 |  | |  |
| Emotional neglect^1^ | -0.38 | | | | 0.92 | | -2.13 - 1.49 | | | | -0.04 | 0.681 |  | |  |
| Emotional abuse^1^ | 0.17 | | | | 0.63 | | -1.09 - 1.38 | | | | 0.02 | 0.788 |  | |  |
| Physical neglect^1^ | 0.03 | | | | 1.01 | | -1.98 - 1.93 | | | | <0.01 | 0.979 |  | |  |
| Physical abuse^1^ | -0.38 | | | | 0.57 | | -1.44 - 0.77 | | | | -0.05 | 0.500 |  | |  |
| Sexual abuse^1^ | -0.46 | | | | 0.51 | | -1.46 - 0.53 | | | | -0.08 | 0.369 |  | |  |
| Discrimination | 0.01 | | | | 0.01 | | -0.02 - 0.02 | | | | 0.04 | 0.648 |  | |  |
| Social exclusion | **-0.04** | | | | 0.01 | | -0.06 - -0.03 | | | | -0.55 | <0.001 |  | |  |
| PTSD-related event^1^ | -0.39 | | | | 0.45 | | -1.25 - 0.47 | | | | -0.08 | 0.408 |  | |  |
|  |  | | | |  | |  | | | |  |  |  | |  |
|  |  | | | |  | |  | | | |  |  |  | |  |
| **Predictor** | ***B*** | | | ***SE*** | **95% *CI*** | | | | **β** | | | ***p*** | ***p (BH)*** | |  |
| **Satisfaction with life** | | | | | | | | | | | | |  | |  |
| **Direct effects** |  | |  | | |  | | | |  | |  |  | |  |
| Cognitive-focused | **0.20** | | 0.02 | | | 0.16 - 0.24 | | | | 0.23 | | <0.001 | <0.001 | |  |
| Emotional-focused | **1.63** | | 0.13 | | | 1.37 - 1.91 | | | | 0.37 | | <0.001 | <0.001 | |  |
| Social-focused | **0.07** | | 0.01 | | | 0.04 - 0.09 | | | | 0.11 | | <0.001 | <0.001 | |  |
| Occupational CLE^1^ | -0.26 | | 0.41 | | | -1.07 - 0.55 | | | | -0.05 | | 0.498 | 0.729 | |  |
| Health-related CLE^1^ | -0.07 | | 0.23 | | | -0.53 - 0.36 | | | | -0.02 | | 0.750 | 0.918 | |  |
| Social CLE^1^ | 0.26 | | 0.27 | | | -0.26 - 0.79 | | | | 0.07 | | 0.328 | 0.563 | |  |
| Emotional neglect^1^ | -1.00 | | 0.59 | | | -2.14 - 0.13 | | | | -0.12 | | 0.084 | 0.248 | |  |
| Emotional abuse^1^ | 0.30 | | 0.43 | | | -0.51 - 1.16 | | | | 0.06 | | 0.488 | 0.729 | |  |
| Physical neglect^1^ | -0.27 | | 0.47 | | | -1.18 - 0.69 | | | | -0.04 | | 0.551 | 0.751 | |  |
| Physical abuse^1^ | -1.32 | | 0.76 | | | -2.87 - 0.15 | | | | -0.14 | | 0.084 | 0.248 | |  |
| Sexual abuse^1^ | 0.64 | | 0.52 | | | -0.40 - 1.66 | | | | 0.10 | | 0.205 | 0.424 | |  |
| Discrimination | 0.01 | | 0.01 | | | -0.01 - 0.03 | | | | 0.06 | | 0.422 | 0.666 | |  |
| Social exclusion | **-0.03** | | 0.01 | | | -0.04 - -0.01 | | | | -0.34 | | <0.001 | <0.001 | |  |
| PTSD-related event^1^ | -0.36 | | 0.36 | | | -1.05 - 0.38 | | | | -0.08 | | 0.322 | 0.563 | |  |
| Age | **-0.05** | | 0.01 | | | -0.07 - -0.02 | | | | -0.06 | | <0.001 | 0.001 | |  |
| Sex (ref. male) | **0.25** | | 0.07 | | | 0.12 - 0.39 | | | | 0.06 | | <0.001 | 0.001 | |  |
| Medium education (ref. low) | **0.62** | | 0.27 | | | 0.11 - 1.15 | | | | 0.12 | | 0.020 | 0.063 | |  |
| High education (ref. low) | 0.35 | | 0.26 | | | -0.15 - 0.88 | | | | 0.08 | | 0.177 | 0.393 | |  |
| In Education (ref. low) | 0.30 | | 0.28 | | | -0.23 - 0.87 | | | | 0.04 | | 0.286 | 0.554 | |  |
| **Moderation effects** |  | |  | | |  | | | |  | |  |  | |  |
| Occupational CLE x SOC | 0.04 | | 0.04 | | | -0.04 - 0.12 | | | | 0.07 | | 0.338 | 0.843 | |  |
| Health-related CLE x SOC | 0.00 | | 0.02 | | | -0.04 - 0.05 | | | | <0.01 | | 0.970 | 0.970 | |  |
| Social CLE x SOC | -0.02 | | 0.03 | | | -0.07 - 0.04 | | | | -0.05 | | 0.518 | 0.873 | |  |
| Emotional neglect x SOC | 0.10 | | 0.07 | | | -0.04 - 0.23 | | | | 0.09 | | 0.160 | 0.588 | |  |
| Emotional abuse x SOC | -0.05 | | 0.05 | | | -0.14 - 0.04 | | | | -0.08 | | 0.310 | 0.843 | |  |
| Physical neglect x SOC | 0.02 | | 0.05 | | | -0.08 - 0.12 | | | | 0.03 | | 0.636 | 0.883 | |  |
| Physical abuse x SOC | 0.16 | | 0.08 | | | -0.01 - 0.33 | | | | 0.14 | | 0.074 | 0.487 | |  |
| Sexual abuse x SOC | -0.04 | | 0.05 | | | -0.15 - 0.07 | | | | -0.06 | | 0.460 | 0.843 | |  |
| Discrimination x SOC | 0.00 | | <0.01 | | | 0.00 - 0.00 | | | | -0.03 | | 0.696 | 0.883 | |  |
| Social exclusion x SOC | **0.00** | | <0.01 | | | 0.00 - 0.00 | | | | 0.20 | | 0.004 | 0.061 | |  |
| PTSD-related event x SOC | 0.04 | | 0.04 | | | -0.04 - 0.11 | | | | 0.07 | | 0.336 | 0.843 | |  |
| **Total effects** |  | |  | | |  | | | |  | |  |  | |  |
| Occupational CLE^1^ | -0.21 | | 0.41 | | | -1.03 - 0.61 | | | | -0.04 | | 0.599 |  | |  |
| Health-related CLE^1^ | -0.09 | | 0.23 | | | -0.54 - 0.36 | | | | -0.03 | | 0.702 |  | |  |
| Social CLE^1^ | 0.28 | | 0.27 | | | -0.27 - 0.83 | | | | 0.07 | | 0.314 |  | |  |
| Emotional neglect^1^ | **-1.19** | | 0.59 | | | -2.34 - -0.06 | | | | -0.14 | | 0.049 |  | |  |
| Emotional abuse^1^ | 0.53 | | 0.52 | | | -0.52 - 1.53 | | | | 0.08 | | 0.295 |  | |  |
| Physical neglect^1^ | -1.30 | | 0.77 | | | -2.84 - 0.12 | | | | -0.13 | | 0.095 |  | |  |
| Physical abuse^1^ | -0.29 | | 0.47 | | | -1.19 - 0.66 | | | | -0.04 | | 0.521 |  | |  |
| Sexual abuse^1^ | -0.08 | | 0.44 | | | -0.92 - 0.79 | | | | -0.02 | | 0.861 |  | |  |
| Discrimination | 0.00 | | 0.01 | | | -0.02 - 0.02 | | | | 0.01 | | 0.942 |  | |  |
| Social exclusion | **-0.04** | | 0.01 | | | -0.05 - -0.03 | | | | -0.58 | | <.001 |  | |  |
| PTSD-related event^1^ | -0.47 | | 0.37 | | | -1.17 - 0.25 | | | | -0.10 | | 0.202 |  | |  |
|  |  | |  | | |  | | | |  | |  |  | |  |
|  |  | |  | | |  | | | |  | |  |  | |  |
|  |  | |  | | |  | | | |  | |  |  | |  |
|  |  | |  | | |  | | | |  | |  |  | |  |
| **Predictor** | | ***B*** | | | | ***SE*** | | **95% *CI*** | | | **β** | ***p*** | | ***p (BH)*** | |
| ***Internalizing symptoms*** | | | | | | | | | | | | |  | |  |
| **Direct effects** |  | | | |  | |  | | | |  |  |  | |  |
| Cognitive-focused | **-0.01** | | | | <0.01 | | -0.01 - -0.01 | | | | -0.12 | <0.001 | <0.001 | |  |
| Emotional-focused | **-0.27** | | | | 0.01 | | -0.30 - -0.24 | | | | -0.61 | <0.001 | <0.001 | |  |
| Social-focused | **0.00** | | | | <0.01 | | -0.01 - 0.00 | | | | -0.05 | 0.002 | 0.006 | |  |
| Occupational CLE^1^ | 0.02 | | | | 0.03 | | -0.04 - 0.08 | | | | 0.04 | 0.478 | 0.697 | |  |
| Health-related CLE^1^ | 0.03 | | | | 0.02 | | -0.02 - 0.07 | | | | 0.09 | 0.187 | 0.382 | |  |
| Social CLE^1^ | 0.03 | | | | 0.02 | | -0.01 - 0.08 | | | | 0.09 | 0.160 | 0.367 | |  |
| Emotional neglect^1^ | 0.02 | | | | 0.05 | | -0.08 - 0.12 | | | | 0.02 | 0.737 | 0.907 | |  |
| Emotional abuse^1^ | -0.05 | | | | 0.04 | | -0.13 - 0.02 | | | | -0.11 | 0.140 | 0.329 | |  |
| Physical neglect^1^ | 0.07 | | | | 0.04 | | -0.02 - 0.15 | | | | 0.10 | 0.128 | 0.310 | |  |
| Physical abuse^1^ | 0.10 | | | | 0.06 | | -0.03 - 0.21 | | | | 0.10 | 0.103 | 0.264 | |  |
| Sexual abuse^1^ | 0.00 | | | | 0.05 | | -0.09 - 0.10 | | | | 0.01 | 0.923 | 0.972 | |  |
| Discrimination | 0.00 | | | | <0.01 | | 0.00 - 0.00 | | | | -0.02 | 0.835 | 0.937 | |  |
| Social exclusion | **0.00** | | | | 0.00 | | 0.00 - 0.00 | | | | 0.22 | 0.002 | 0.006 | |  |
| PTSD-related event^1^ | 0.03 | | | | 0.03 | | -0.04 - 0.09 | | | | 0.05 | 0.427 | 0.645 | |  |
| Age | **0.01** | | | | <0.01 | | 0.00 - 0.01 | | | | 0.07 | <0.001 | <0.001 | |  |
| Sex (ref. male) | **0.02** | | | | 0.01 | | 0.00 - 0.03 | | | | 0.04 | 0.009 | 0.032 | |  |
| Medium education (ref. low) | 0.00 | | | | 0.02 | | -0.04 - 0.05 | | | | 0.01 | 0.913 | 0.972 | |  |
| High education (ref. low) | 0.02 | | | | 0.02 | | -0.02 - 0.07 | | | | 0.06 | 0.265 | 0.494 | |  |
| In Education (ref. low) | 0.03 | | | | 0.02 | | -0.02 - 0.07 | | | | 0.05 | 0.193 | 0.382 | |  |
| **Moderation effects** |  | | | |  | |  | | | |  |  |  | |  |
| Occupational CLE x SOC | 0.00 | | | | <0.01 | | -0.01 - 0.00 | | | | -0.07 | 0.239 | 0.702 | |  |
| Health-related CLE x SOC | 0.00 | | | | <0.01 | | -0.01 - 0.00 | | | | -0.06 | 0.364 | 0.789 | |  |
| Social CLE x SOC | 0.00 | | | | <0.01 | | -0.01 - 0.00 | | | | -0.09 | 0.131 | 0.504 | |  |
| Emotional neglect x SOC | 0.00 | | | | 0.01 | | -0.01 - 0.01 | | | | 0.02 | 0.730 | 0.908 | |  |
| Emotional abuse x SOC | 0.01 | | | | <0.01 | | 0.00 - 0.02 | | | | 0.13 | 0.058 | 0.406 | |  |
| Physical neglect x SOC | -0.01 | | | | 0.01 | | -0.02 - 0.00 | | | | -0.10 | 0.088 | 0.428 | |  |
| Physical abuse x SOC | **-0.01** | | | | 0.01 | | -0.03 - 0.00 | | | | -0.12 | 0.032 | 0.356 | |  |
| Sexual abuse x SOC | 0.00 | | | | 0.01 | | -0.01 - 0.01 | | | | 0.01 | 0.863 | 0.969 | |  |
| Discrimination x SOC | 0.00 | | | | 0.00 | | 0.00 - 0.00 | | | | -0.01 | 0.946 | 0.970 | |  |
| Social exclusion x SOC | 0.00 | | | | 0.00 | | 0.00 - 0.00 | | | | -0.11 | 0.071 | 0.406 | |  |
| PTSD-related event x SOC | 0.00 | | | | <0.01 | | -0.01 - 0.00 | | | | -0.04 | 0.532 | 0.836 | |  |
| **Total effects** |  | | | |  | |  | | | |  |  |  | |  |
| Occupational CLE^1^ | 0.02 | | | | 0.03 | | -0.05 - 0.08 | | | | 0.04 | 0.570 |  | |  |
| Health-related CLE^1^ | 0.03 | | | | 0.02 | | -0.01 - 0.07 | | | | 0.10 | 0.123 |  | |  |
| Social CLE^1^ | 0.04 | | | | 0.02 | | -0.01 - 0.09 | | | | 0.10 | 0.107 |  | |  |
| Emotional neglect^1^ | 0.04 | | | | 0.05 | | -0.06 - 0.14 | | | | 0.04 | 0.487 |  | |  |
| Emotional abuse^1^ | 0.03 | | | | 0.05 | | -0.07 - 0.12 | | | | 0.04 | 0.581 |  | |  |
| Physical neglect^1^ | 0.10 | | | | 0.06 | | -0.02 - 0.22 | | | | 0.10 | 0.091 |  | |  |
| Physical abuse^1^ | 0.06 | | | | 0.04 | | -0.02 - 0.15 | | | | 0.10 | 0.138 |  | |  |
| Sexual abuse^1^ | 0.00 | | | | 0.04 | | -0.08 - 0.07 | | | | <-0.01 | 0.970 |  | |  |
| Discrimination | 0.00 | | | | <0.01 | | 0.00 - 0.00 | | | | 0.07 | 0.325 |  | |  |
| Social exclusion | **0.00** | | | | 0.00 | | 0.00 - 0.01 | | | | 0.49 | <0.001 |  | |  |
| PTSD-related event ^1^ | 0.04 | | | | 0.03 | | -0.02 - 0.10 | | | | 0.09 | 0.200 |  | |  |
|  |  | | | |  | |  | | | |  |  |  | |  |
|  |  | | | |  | |  | | | |  |  |  | |  |
|  |  | | | |  | |  | | | |  |  |  | |  |
|  |  | | | |  | |  | | | |  |  |  | |  |
| **Predictor** | ***B*** | | | | ***SE*** | | **95% *CI*** | | | | **β** | ***p*** |  | |  |
| ***Externalizing personality*** | | | | | | | | | | | | |  | |  |
| **Direct** **effects** |  | | | |  | |  | | | |  |  |  | |  |
| Cognitive-focused | **-0.01** | | | | <0.01 | | -0.01 - 0.00 | | | | -0.09 | <0.001 | 0.001 | |  |
| Emotional-focused | **-0.17** | | | | 0.01 | | -0.20 - -0.15 | | | | -0.50 | <0.001 | <0.001 | |  |
| Social-focused | 0.00 | | | | <0.01 | | 0.00 - 0.00 | | | | 0.01 | 0.688 | 0.871 | |  |
| Occupational CLE^1^ | 0.04 | | | | 0.04 | | -0.03 - 0.11 | | | | 0.09 | 0.327 | 0.529 | |  |
| Health-related CLE^1^ | 0.00 | | | | 0.02 | | -0.05 - 0.04 | | | | -0.01 | 0.911 | 0.961 | |  |
| Social CLE^1^ | 0.03 | | | | 0.03 | | -0.02 - 0.08 | | | | 0.10 | 0.242 | 0.457 | |  |
| Emotional neglect^1^ | 0.02 | | | | 0.07 | | -0.13 - 0.16 | | | | 0.02 | 0.837 | 0.935 | |  |
| Emotional abuse^1^ | 0.01 | | | | 0.04 | | -0.07 - 0.08 | | | | 0.02 | 0.860 | 0.935 | |  |
| Physical neglect^1^ | 0.04 | | | | 0.05 | | -0.06 - 0.13 | | | | 0.07 | 0.424 | 0.638 | |  |
| Physical abuse^1^ | 0.10 | | | | 0.07 | | -0.04 - 0.25 | | | | 0.13 | 0.164 | 0.374 | |  |
| Sexual abuse^1^ | 0.02 | | | | 0.05 | | -0.07 - 0.12 | | | | 0.05 | 0.624 | 0.821 | |  |
| Discrimination | 0.00 | | | | <0.01 | | 0.00 - 0.00 | | | | -0.14 | 0.103 | 0.271 | |  |
| Social exclusion | 0.00 | | | | 0.00 | | 0.00 - 0.00 | | | | -0.03 | 0.754 | 0.909 | |  |
| PTSD-related event ^1^ | 0.04 | | | | 0.03 | | -0.03 - 0.11 | | | | 0.11 | 0.223 | 0.428 | |  |
| Age | **0.00** | | | | <0.01 | | -0.01 - 0.00 | | | | -0.05 | 0.011 | 0.040 | |  |
| Sex (ref. male) | **-0.08** | | | | 0.01 | | -0.09 - -0.06 | | | | -0.23 | <0.001 | <0.001 | |  |
| Medium eductaion (ref. low) | -0.04 | | | | 0.02 | | -0.09 - 0.00 | | | | -0.11 | 0.067 | 0.211 | |  |
| High education (ref. low) | -0.04 | | | | 0.02 | | -0.09 - 0.00 | | | | -0.13 | 0.061 | 0.198 | |  |
| In Education (ref. low) | -0.03 | | | | 0.03 | | -0.08 - 0.02 | | | | -0.05 | 0.274 | 0.498 | |  |
| **Moderation effects** |  | | | |  | |  | | | |  |  |  | |  |
| Occupational CLE x SOC | 0.00 | | | | <0.01 | | -0.01 - 0.00 | | | | -0.07 | 0.424 | 0.815 | |  |
| Health-related CLE x SOC | 0.00 | | | | <0.01 | | 0.00 - 0.01 | | | | 0.03 | 0.719 | 0.889 | |  |
| Social CLE x SOC | 0.00 | | | | <0.01 | | -0.01 - 0.00 | | | | -0.07 | 0.394 | 0.815 | |  |
| Emotional neglect x SOC | 0.00 | | | | 0.01 | | -0.02 - 0.02 | | | | <-0.01 | 0.990 | 0.990 | |  |
| Emotional abuse x SOC | 0.00 | | | | <0.01 | | -0.01 - 0.01 | | | | 0.03 | 0.724 | 0.889 | |  |
| Physical neglect x SOC | 0.00 | | | | 0.01 | | -0.01 - 0.01 | | | | -0.07 | 0.425 | 0.815 | |  |
| Physical abuse x SOC | -0.01 | | | | 0.01 | | -0.03 - 0.01 | | | | -0.09 | 0.326 | 0.815 | |  |
| Sexual abuse x SOC | 0.00 | | | | 0.01 | | -0.01 - 0.01 | | | | -0.03 | 0.719 | 0.889 | |  |
| Discrimination x SOC | **0.00** | | | | 0.00 | | 0.00 - 0.00 | | | | 0.17 | 0.039 | 0.426 | |  |
| Social exclusion x SOC | 0.00 | | | | 0.00 | | 0.00 - 0.00 | | | | -0.05 | 0.552 | 0.850 | |  |
| PTSD-related event x SOC | 0.00 | | | | <0.01 | | -0.01 - 0.00 | | | | -0.09 | 0.294 | 0.815 | |  |
| **Total effects** |  | | | |  | |  | | | |  |  |  | |  |
| Occupational CLE^1^ | 0.04 | | | | 0.04 | | -0.04 - 0.11 | | | | 0.08 | 0.321 |  | |  |
| Health-related CLE^1^ | 0.00 | | | | 0.02 | | -0.04 - 0.05 | | | | <0.01 | 0.969 |  | |  |
| Social CLE^1^ | 0.04 | | | | 0.03 | | -0.02 - 0.09 | | | | 0.11 | 0.174 |  | |  |
| Emotional neglect^1^ | 0.02 | | | | 0.07 | | -0.12 - 0.17 | | | | 0.04 | 0.740 |  | |  |
| Emotional abuse^1^ | 0.04 | | | | 0.05 | | -0.05 - 0.13 | | | | 0.08 | 0.417 |  | |  |
| Physical neglect^1^ | 0.11 | | | | 0.07 | | -0.04 - 0.25 | | | | 0.13 | 0.143 |  | |  |
| Physical abuse^1^ | 0.04 | | | | 0.05 | | -0.06 - 0.13 | | | | 0.07 | 0.449 |  | |  |
| Sexual abuse^1^ | 0.04 | | | | 0.04 | | -0.04 - 0.12 | | | | 0.10 | 0.290 |  | |  |
| Discrimination | 0.00 | | | | <0.01 | | 0.00 - 0.00 | | | | -0.06 | 0.433 |  | |  |
| Social exclusion | **0.00** | | | | 0.00 | | 0.00 - 0.00 | | | | 0.18 | 0.026 |  | |  |
| PTSD-related event ^1^ | 0.05 | | | | 0.03 | | -0.02 - 0.12 | | | | 0.14 | 0.125 |  | |  |
|  |  | | | |  | |  | | | |  |  |  | |  |
|  |  | | | |  | |  | | | |  |  |  | |  |
|  |  | | | |  | |  | | | |  |  |  | |  |
|  |  | | | |  | |  | | | |  |  |  | |  |
| **Predictor** | ***B*** | | | | ***SE*** | | **95% *CI*** | | | | **β** | ***p*** | ***p (BH)*** | |  |
| ***Substance use*** | | | | | | | | | | | | |  | |  |
| **Direct effects** |  | | | |  | |  | | | |  |  |  | |  |
| Cognitive-focused | **0.00** | | | | <0.01 | | -0.01 - 0.00 | | | | -0.07 | 0.004 | 0.016 | |  |
| Emotional-focused | **-0.10** | | | | 0.01 | | -0.12 - -0.08 | | | | -0.35 | <0.001 | <0.001 | |  |
| Social-focused | 0.00 | | | | <0.01 | | 0.00 - 0.00 | | | | 0.04 | 0.102 | 0.258 | |  |
| Occupational CLE^1^ | 0.06 | | | | 0.04 | | -0.01 - 0.13 | | | | 0.17 | 0.085 | 0.244 | |  |
| Health-related CLE^1^ | 0.00 | | | | 0.02 | | -0.04 - 0.04 | | | | -0.01 | 0.950 | 0.983 | |  |
| Social CLE^1^ | 0.02 | | | | 0.03 | | -0.04 - 0.07 | | | | 0.06 | 0.551 | 0.743 | |  |
| Emotional neglect^1^ | 0.01 | | | | 0.06 | | -0.11 - 0.12 | | | | 0.02 | 0.843 | 0.930 | |  |
| Emotional abuse^1^ | -0.05 | | | | 0.04 | | -0.12 - 0.02 | | | | -0.15 | 0.186 | 0.373 | |  |
| Physical neglect^1^ | -0.02 | | | | 0.05 | | -0.11 - 0.06 | | | | -0.05 | 0.557 | 0.743 | |  |
| Physical abuse^1^ | 0.04 | | | | 0.07 | | -0.10 - 0.19 | | | | 0.05 | 0.609 | 0.795 | |  |
| Sexual abuse^1^ | 0.06 | | | | 0.05 | | -0.05 - 0.16 | | | | 0.13 | 0.294 | 0.497 | |  |
| Discrimination | 0.00 | | | | <0.01 | | 0.00 - 0.00 | | | | 0.07 | 0.557 | 0.743 | |  |
| Social exclusion | **0.00** | | | | 0.00 | | 0.00 - 0.00 | | | | -0.26 | 0.005 | 0.021 | |  |
| PTSD-related event^1^ | 0.05 | | | | 0.03 | | -0.01 - 0.12 | | | | 0.16 | 0.118 | 0.279 | |  |
| Age | **0.00** | | | | <0.01 | | 0.00 - 0.01 | | | | 0.06 | 0.006 | 0.024 | |  |
| Sex (ref. male) | **-0.05** | | | | 0.01 | | -0.06 - -0.04 | | | | -0.17 | <0.001 | <0.001 | |  |
| Medium education (ref. low) | -0.03 | | | | 0.03 | | -0.09 - 0.02 | | | | -0.09 | 0.233 | 0.430 | |  |
| High education (ref. low) | **-0.05** | | | | 0.03 | | -0.11 - 0.00 | | | | -0.18 | 0.048 | 0.153 | |  |
| In Education (ref. low) | **-0.07** | | | | 0.03 | | -0.13 - -0.02 | | | | -0.15 | 0.015 | 0.053 | |  |
| **Moderation effects** |  | | | |  | |  | | | |  |  |  | |  |
| Occupational CLE x SOC | -0.01 | | | | <0.01 | | -0.01 - 0.00 | | | | -0.13 | 0.155 | 0.661 | |  |
| Health-related CLE x SOC | 0.00 | | | | <0.01 | | 0.00 - 0.00 | | | | 0.02 | 0.855 | 0.982 | |  |
| Social CLE x SOC | 0.00 | | | | <0.01 | | -0.01 - 0.01 | | | | -0.01 | 0.954 | 0.985 | |  |
| Emotional neglect x SOC | 0.00 | | | | 0.01 | | -0.01 - 0.01 | | | | <-0.01 | 0.970 | 0.985 | |  |
| Emotional abuse x SOC | 0.01 | | | | <0.01 | | 0.00 - 0.01 | | | | 0.12 | 0.234 | 0.798 | |  |
| Physical neglect x SOC | 0.00 | | | | 0.01 | | -0.01 - 0.01 | | | | 0.08 | 0.406 | 0.798 | |  |
| Physical abuse x SOC | 0.00 | | | | 0.01 | | -0.02 - 0.01 | | | | -0.03 | 0.774 | 0.928 | |  |
| Sexual abuse x SOC | -0.01 | | | | 0.01 | | -0.02 - 0.01 | | | | -0.11 | 0.306 | 0.798 | |  |
| Discrimination x SOC | 0.00 | | | | 0.00 | | 0.00 - 0.00 | | | | -0.09 | 0.406 | 0.798 | |  |
| Social exclusion x SOC | 0.00 | | | | 0.00 | | 0.00 - 0.00 | | | | 0.11 | 0.190 | 0.798 | |  |
| PTSD-related event x SOC | 0.00 | | | | <0.01 | | -0.01 - 0.00 | | | | -0.09 | 0.347 | 0.798 | |  |
| **Total effects** |  | | | |  | |  | | | |  |  |  | |  |
| Occupational CLE^1^ | 0.06 | | | | 0.04 | | -0.02 - 0.13 | | | | 0.17 | 0.088 |  | |  |
| Health-related CLE^1^ | 0.00 | | | | 0.02 | | -0.04 - 0.04 | | | | <0.01 | 0.979 |  | |  |
| Social CLE^1^ | 0.02 | | | | 0.03 | | -0.03 - 0.07 | | | | 0.07 | 0.468 |  | |  |
| Emotional neglect^1^ | 0.02 | | | | 0.06 | | -0.10 - 0.13 | | | | 0.03 | 0.786 |  | |  |
| Emotional abuse^1^ | 0.07 | | | | 0.05 | | -0.04 - 0.17 | | | | 0.15 | 0.215 |  | |  |
| Physical neglect^1^ | 0.04 | | | | 0.07 | | -0.10 - 0.19 | | | | 0.06 | 0.589 |  | |  |
| Physical abuse^1^ | -0.03 | | | | 0.05 | | -0.12 - 0.06 | | | | -0.06 | 0.582 |  | |  |
| Sexual abuse^1^ | -0.03 | | | | 0.04 | | -0.10 - 0.04 | | | | -0.09 | 0.417 |  | |  |
| Discrimination | 0.00 | | | | <0.01 | | 0.00 - 0.00 | | | | 0.12 | 0.298 |  | |  |
| Social exclusion | 0.00 | | | | 0.00 | | 0.00 - 0.00 | | | | -0.12 | 0.185 |  | |  |
| PTSD-related event ^1^ | 0.06 | | | | 0.03 | | -0.01 - 0.13 | | | | 0.18 | 0.079 |  | |  |

*Notes.* ^1^ reference = yes (1), CLE = critical life events, SOC = social support, PTSD sym. = post-traumatic stress disorder symptoms. Significant effects at p < .01 based on Benjamini-Hochberg adjusted are highlighted in bold.

**Table S6.**

Cross-sectional indirect associations via transdiagnostic process variables (cognitive-, emotional- and social-focused processes) linking potentially traumatic event indicators with mental health outcomes (N=3,051)

| Indirect effects | | B | SE | 95% *CI* | β | p | p (BH) | SIE |  | B | | SE | | | 95% *CI* | β | p | p (BH) | SIE | |
| --- | --- | --- | --- | --- | --- | --- | --- | --- | --- | --- | --- | --- | --- | --- | --- | --- | --- | --- | --- | --- |
|  | Resilience | | | | | | | |  |  | | | Meaning of Life | | | | | | | |
| Occupational CLE → cm | | 0.01 | 0.01 | -0.01 - 0.04 | 0.01 | 0.265 | 0.625 | 0.004 |  | 0.04 | | 0.04 | | | -0.03 - 0.10 | 0.01 | 0.266 | 0.488 | 0.001 | |
| Occupational CLE → em | | 0.00 | 0.01 | -0.01 - 0.02 | <0.01 | 0.758 | 0.939 | 0.001 |  | 0.01 | | 0.03 | | | -0.05 - 0.07 | <0.01 | 0.752 | 0.896 | <.001 | |
| Occupational CLE → sm | | 0.00 | <0.01 | 0.00 - 0.00 | 0.00 | 0.850 | 0.939 | <.001 |  | 0.02 | | 0.01 | | | 0.00 - 0.05 | <0.01 | 0.094 | 0.258 | 0.001 | |
| Health-related CLE → cm | | 0.01 | 0.01 | 0.00 - 0.03 | 0.01 | 0.131 | 0.359 | 0.008 |  | 0.03 | | 0.02 | | | -0.01 - 0.08 | 0.01 | 0.132 | 0.298 | <.001 | |
| Health-related CLE → em | | -0.01 | 0.01 | -0.02 - 0.00 | -0.01 | 0.142 | 0.359 | -0.005 |  | -0.03 | | 0.02 | | | -0.06 - 0.01 | -0.01 | 0.149 | 0.298 | -0.002 | |
| Health-related CLE → sm | | 0.00 | <0.01 | 0.00 - 0.00 | 0.00 | 0.957 | 0.957 | <.001 |  | 0.00 | | 0.01 | | | -0.02 - 0.01 | 0.00 | 0.816 | 0.896 | 0.003 | |
| Social CLE → cm | | 0.03 | 0.01 | 0.01 - 0.04 | 0.02 | 0.007 | 0.044 | 0.001 |  | 0.07 | | 0.03 | | | 0.02 - 0.12 | 0.02 | 0.009 | 0.046 | 0.004 | |
| Social CLE → em | | -0.01 | 0.01 | -0.02 - 0.00 | -0.01 | 0.053 | 0.219 | -0.005 |  | -0.05 | | 0.02 | | | -0.09 - 0.00 | -0.01 | 0.040 | 0.148 | -0.002 | |
| Social CLE → sm | | 0.00 | <0.01 | 0.00 - 0.01 | 0.00 | 0.831 | 0.939 | <.001 |  | 0.03 | | 0.01 | | | 0.01 - 0.05 | 0.01 | 0.004 | 0.027 | 0.002 | |
| Emotional neglect→ cm | | 0.00 | 0.02 | -0.08 - 0.01 | -0.01 | 0.137 | 0.359 | -0.003 |  | 0.00 | | 0.07 | | | -0.23 - 0.03 | -0.01 | 0.147 | 0.298 | -0.001 | |
| Emotional neglect→ em | | 0.00 | 0.01 | -0.04 - 0.01 | -0.01 | 0.286 | 0.630 | -0.001 |  | 0.00 | | 0.05 | | | -0.16 - 0.05 | -0.01 | 0.285 | 0.497 | -0.001 | |
| Emotional neglect→ sm | | 0.00 | 0.01 | -0.01 - 0.01 | 0.00 | 0.828 | 0.939 | -0.003 |  | 0.00 | | 0.03 | | | -0.12 - -0.02 | -0.01 | 0.011 | 0.051 | -0.001 | |
| Emotional abuse → cm | | 0.02 | 0.02 | -0.03 - 0.04 | <0.01 | 0.729 | 0.939 | 0.001 |  | 0.05 | | 0.05 | | | -0.08 - 0.11 | <0.01 | 0.736 | 0.896 | <.001 | |
| Emotional abuse → em | | -0.01 | 0.01 | -0.05 - -0.01 | -0.01 | 0.014 | 0.078 | -0.005 |  | -0.04 | | 0.04 | | | -0.19 - -0.03 | -0.02 | 0.008 | 0.045 | -0.002 | |
| Emotional abuse → sm | | 0.00 | <0.01 | -0.01 - 0.01 | 0.00 | 0.855 | 0.939 | <.001 |  | 0.04 | | 0.02 | | | -0.01 - 0.05 | <0.01 | 0.156 | 0.303 | <.001 | |
| Physical neglect → cm | | -0.02 | 0.02 | -0.04 - 0.02 | <-0.01 | 0.533 | 0.939 | -0.002 |  | -0.07 | | 0.05 | | | -0.13 - 0.07 | <-0.01 | 0.527 | 0.793 | -0.001 | |
| Physical neglect → em | | -0.06 | 0.01 | -0.02 - 0.03 | <0.01 | 0.677 | 0.939 | 0.001 |  | -0.23 | | 0.04 | | | -0.06 - 0.10 | <0.01 | 0.632 | 0.851 | <.001 | |
| Physical neglect → sm | | 0.00 | <0.01 | -0.01 - 0.00 | 0.00 | 0.847 | 0.939 | <.001 |  | -0.03 | | 0.02 | | | -0.06 - 0.00 | <-0.01 | 0.098 | 0.258 | -0.001 | |
| Physical abuse→ cm | | 0.00 | 0.03 | -0.04 - 0.07 | <0.01 | 0.541 | 0.939 | 0.001 |  | -0.01 | | 0.07 | | | -0.10 - 0.19 | <0.01 | 0.553 | 0.793 | -0.004 | |
| Physical abuse→ em | | 0.00 | 0.02 | -0.04 - 0.02 | <-0.01 | 0.495 | 0.939 | -0.001 |  | -0.01 | | 0.06 | | | -0.16 - 0.07 | <-0.01 | 0.298 | 0.782 | <.001 | |
| Physical abuse→ sm | | 0.00 | 0.00 | -0.01 - 0.01 | 0.00 | 0.841 | 0.939 | <.001 |  | 0.00 | | 0.03 | | | -0.01 - 0.09 | <0.01 | 0.096 | 0.258 | <.001 | |
| Sexual abuse → cm | | -0.03 | 0.01 | -0.05 - 0.00 | -0.01 | 0.089 | 0.328 | -0.007 |  | -0.10 | | 0.04 | | | -0.15 - 0.01 | -0.01 | 0.086 | 0.258 | -0.002 | |
| Sexual abuse → em | | -0.02 | 0.01 | -0.09 - -0.04 | -0.03 | <.001 | <.001 | -0.017 |  | -0.06 | | 0.04 | | | -0.32 - -0.15 | -0.04 | <.001 | 0.156 | -0.007 | |
| Sexual abuse → sm | | 0.00 | 0.00 | -0.01 - 0.00 | 0.00 | 0.838 | 0.939 | <.001 |  | -0.07 | | 0.01 | | | -0.06 - -0.01 | -0.01 | 0.043 | 0.045 | -0.001 | |
| Discrimination→ cm | | 0.01 | 0.02 | -0.01 - 0.00 | -0.01 | 0.133 | 0.359 | -0.003 |  | 0.02 | | <0.01 | | | 0.00 - 0.00 | -0.01 | 0.125 | 0.298 | -0.001 | |
| Discrimination→ em | | -0.03 | 0.00 | -0.05 - -0.01 | -0.03 | <.001 | <.001 | -0.006 |  | -0.11 | | <0.01 | | | -0.01 - -0.01 | -0.04 | <.001 | <.001 | -0.003 | |
| Discrimination→ sm | | 0.00 | <0.01 | 0.00 - 0.00 | 0.00 | 0.911 | 0.939 | <.001 |  | 0.02 | | 0.00 | | | 0.00 - 0.00 | <0.01 | 0.576 | 0.799 | <.001 | |
| Social exclusion → cm | | -0.01 | 0.00 | -0.04 - 0.02 | -0.09 | <.001 | <.001 | -0.033 |  | -0.03 | | <0.01 | | | -0.01 - -0.01 | -0.09 | <.001 | <.001 | -0.011 | |
| Social exclusion → em | | 0.00 | 0.01 | -0.03 - 0.01 | -0.07 | <.001 | <.001 | -0.027 |  | 0.02 | | <0.01 | | | -0.01 - -0.01 | -0.10 | <.001 | <.001 | -0.012 | |
| Social exclusion → sm | | 0.00 | <0.01 | -0.01 - 0.00 | 0.00 | 0.824 | 0.939 | -0.001 |  | -0.03 | | <0.01 | | | -0.01 - -0.01 | -0.03 | <.001 | <.001 | -0.004 | |
| PTSD event → cm | | -0.01 | 0.01 | -0.03 - 0.01 | -0.01 | 0.418 | 0.861 | <.001 |  | -0.03 | | 0.03 | | | -0.10 - 0.04 | -0.01 | 0.412 | 0.689 | <.001 | |
| PTSD event → em | | -0.02 | 0.01 | -0.04 - 0.00 | -0.01 | 0.024 | 0.115 | -0.006 |  | -0.07 | | 0.03 | | | -0.13 - -0.01 | -0.01 | 0.015 | 0.063 | -0.003 | |
| PTSD event → sm | | 0.00 | <0.01 | 0.00 - 0.00 | 0.00 | 0.908 | 0.939 | -0.003 |  | 0.01 | | 0.01 | | | -0.02 - 0.03 | <0.01 | 0.577 | 0.799 | -0.001 | |
|  | Satisfaction with life | | | | | | | |  | |  | | | Internalizing symptoms | | | | | |  |
| Occupational CLE → cm | | 0.03 | 0.02 | -0.02 - 0.07 | 0.01 | 0.266 | 0.455 | 0.001 |  | 0.00 | | <0.01 | | | 0.00 - 0.00 | <-0.01 | 0.279 | 0.455 | -0.005 | |
| Occupational CLE → em | | 0.01 | 0.04 | -0.06 - 0.09 | <0.01 | 0.737 | 0.868 | 0.001 |  | 0.00 | | 0.01 | | | -0.02 - 0.01 | <-0.01 | 0.720 | 0.841 | -0.008 | |
| Occupational CLE → sm | | 0.02 | 0.01 | 0.00 - 0.04 | <0.01 | 0.098 | 0.242 | 0.001 |  | 0.00 | | <0.01 | | | 0.00 - 0.00 | <-0.01 | 0.121 | 0.275 | -0.003 | |
| Health-related CLE → cm | | 0.02 | 0.01 | -0.01 - 0.05 | 0.01 | 0.122 | 0.280 | <.001 |  | 0.00 | | <0.01 | | | 0.00 - 0.00 | <-0.01 | 0.134 | 0.275 | -0.010 | |
| Health-related CLE → em | | -0.04 | 0.02 | -0.09 - 0.01 | -0.01 | 0.158 | 0.289 | -0.003 |  | 0.01 | | <0.01 | | | 0.00 - 0.01 | 0.02 | 0.128 | 0.275 | 0.055 | |
| Health-related CLE → sm | | 0.00 | 0.01 | -0.02 - 0.01 | 0.00 | 0.811 | 0.882 | <.001 |  | 0.00 | | 0.00 | | | 0.00 - 0.00 | 0.00 | 0.812 | 0.875 | 0.001 | |
| Social CLE → cm | | 0.05 | 0.02 | 0.01 - 0.08 | 0.01 | 0.009 | 0.043 | 0.003 |  | 0.00 | | <0.01 | | | 0.00 - 0.00 | -0.01 | 0.012 | 0.049 | -0.015 | |
| Social CLE → em | | -0.06 | 0.03 | -0.12 - -0.01 | -0.02 | 0.036 | 0.126 | -0.004 |  | 0.01 | | 0.01 | | | 0.01 - 0.02 | 0.02 | 0.033 | 0.116 | 0.061 | |
| Social CLE → sm | | 0.03 | 0.01 | 0.01 - 0.05 | 0.01 | 0.003 | 0.018 | 0.002 |  | 0.00 | | <0.01 | | | 0.00 - 0.00 | -0.01 | 0.021 | 0.080 | -0.008 | |
| Emotional neglect→ cm | | -0.06 | 0.04 | -0.15 - 0.02 | -0.01 | 0.148 | 0.284 | -0.001 |  | 0.00 | | 0.00 | | | 0.00 - 0.01 | <0.01 | 0.159 | 0.285 | 0.004 | |
| Emotional neglect→ em | | -0.07 | 0.07 | -0.21 - 0.06 | -0.01 | 0.293 | 0.059 | -0.001 |  | 0.00 | | 0.01 | | | -0.01 - 0.03 | 0.02 | 0.250 | 0.429 | 0.017 | |
| Emotional neglect→ sm | | -0.06 | 0.02 | -0.11 - -0.02 | -0.01 | 0.010 | 0.044 | -0.001 |  | 0.00 | | 0.00 | | | 0.01 - 0.01 | 0.01 | 0.035 | 0.121 | 0.004 | |
| Emotional abuse → cm | | 0.01 | 0.03 | -0.05 - 0.07 | <0.01 | 0.730 | 0.868 | <.001 |  | 0.00 | | <0.01 | | | -0.01 - 0.00 | <-0.01 | 0.739 | 0.841 | -0.002 | |
| Emotional abuse → em | | -0.14 | 0.05 | -0.25 - -0.05 | -0.02 | 0.006 | 0.035 | -0.003 |  | 0.01 | | 0.01 | | | -0.02 - 0.03 | 0.01 | 0.007 | 0.037 | 0.021 | |
| Emotional abuse → sm | | 0.02 | 0.01 | -0.01 - 0.05 | 0.00 | 0.172 | 0.310 | 0.001 |  | 0.00 | | <0.01 | | | -0.01 - 0.00 | <-0.01 | 0.204 | 0.354 | -0.002 | |
| Physical neglect → cm | | -0.02 | 0.03 | -0.08 - 0.04 | -0.01 | 0.534 | 0.743 | <.001 |  | 0.00 | | <0.01 | | | 0.00 - 0.00 | <0.01 | 0.541 | 0.728 | 0.002 | |
| Physical neglect → em | | 0.03 | 0.05 | -0.08 - 0.12 | <0.01 | 0.631 | 0.792 | 0.001 |  | 0.05 | | 0.01 | | | -0.02 - 0.01 | -0.01 | 0.657 | 0.796 | -0.008 | |
| Physical neglect → sm | | -0.03 | 0.01 | -0.05 - 0.00 | -0.01 | 0.172 | 0.310 | -0.001 |  | 0.00 | | <0.01 | | | 0.00 - 0.00 | <0.01 | 0.120 | 0.275 | 0.003 | |
| Physical abuse→ cm | | 0.03 | 0.05 | -0.07 - 0.12 | <0.01 | 0.534 | 0.743 | <.001 |  | 0.00 | | 0.00 | | | 0.00 - 0.00 | <-0.01 | 0.546 | 0.728 | 0.008 | |
| Physical abuse→ em | | -0.05 | 0.07 | -0.19 - 0.09 | -0.01 | 0.490 | 0.736 | -0.001 |  | 0.00 | | 0.00 | | | -0.01 - 0.00 | <-0.01 | 0.539 | 0.728 | <.001 | |
| Physical abuse→ sm | | 0.04 | 0.02 | -0.01 - 0.09 | <0.01 | 0.110 | 0.265 | <.001 |  | 0.00 | | 0.00 | | | -0.01 - 0.00 | <-0.01 | 0.160 | 0.285 | -0.002 | |
| Sexual abuse → cm | | -0.05 | 0.03 | -0.10 - 0.00 | -0.01 | 0.093 | 0.242 | -0.002 |  | 0.00 | | 0.01 | | | 0.04 - 0.07 | 0.10 | 0.097 | 0.243 | 0.195 | |
| Sexual abuse → em | | -0.31 | 0.05 | -0.40 - -0.22 | -0.06 | <.001 | <.001 | -0.012 |  | 0.01 | | 0.01 | | | 0.04 - 0.07 | 0.10 | <.001 | <.001 | 0.195 | |
| Sexual abuse → sm | | -0.03 | 0.01 | -0.05 - -0.01 | -0.01 | 0.041 | 0.137 | -0.001 |  | 0.00 | | <0.01 | | | 0.00 - 0.03 | <0.01 | 0.089 | 0.243 | 0.005 | |
| Discrimination→ cm | | 0.01 | 0.00 | 0.00 - 0.00 | -0.01 | 0.126 | 0.280 | -0.001 |  | 0.00 | | 0.00 | | | 0.00 - 0.00 | <0.01 | 0.137 | 0.275 | -0.001 | |
| Discrimination→ em | | -0.14 | 0.01 | -0.01 - 0.01 | -0.05 | <.001 | <.001 | -0.004 |  | 0.02 | | 0.00 | | | 0.01 - 0.01 | 0.08 | <.001 | <.001 | 0.072 | |
| Discrimination→ sm | | 0.02 | 0.00 | 0.00 - 0.00 | <0.01 | 0.574 | 0.747 | <.001 |  | 0.00 | | 0.00 | | | 0.00 - 0.00 | 0.00 | 0.603 | 0.750 | -0.001 | |
| Social exclusion → cm | | -0.02 | 0.00 | -0.03 - -0.01 | -0.06 | <.001 | <.001 | -0.009 |  | 0.00 | | <0.01 | | | 0.01 - 0.01 | 0.03 | <.001 | <.001 | 0.043 | |
| Social exclusion → em | | 0.03 | 0.00 | -0.01 - -0.01 | -0.14 | <.001 | <.001 | -0.019 |  | 0.00 | | 0.00 | | | 0.01 - 0.01 | 0.23 | <.001 | <.001 | 0.308 | |
| Social exclusion → sm | | -0.03 | 0.00 | -0.01 - -0.01 | -0.03 | <.001 | <.001 | -0.004 |  | 0.00 | | <0.01 | | | 0.01 - 0.01 | 0.02 | 0.003 | 0.020 | 0.021 | |
| PTSD event → cm | | -0.02 | 0.02 | -0.06 - 0.02 | <-0.01 | 0.423 | 0.655 | 0.001 |  | 0.00 | | <0.01 | | | 0.00 - 0.00 | <0.01 | 0.415 | 0.634 | 0.004 | |
| PTSD event → em | | -0.09 | 0.04 | -0.17 - -0.03 | -0.02 | 0.014 | 0.056 | -0.004 |  | 0.02 | | 0.01 | | | 0.00 - 0.03 | 0.03 | 0.011 | 0.049 | 0.067 | |
| PTSD event → sm | | 0.01 | 0.01 | -0.01 - 0.03 | <0.01 | 0.572 | 0.747 | <.001 |  | 0.00 | | 0.00 | | | 0.00 - 0.00 | <-0.01 | 0.591 | 0.743 | -0.001 | |
|  | Externalizing personality traits | | | | | | | |  |  | | | Substance use | | | | | | | |
| Occupational CLE → cm | | 0.00 | <0.01 | 0.00 - 0.00 | <-0.01 | 0.312 | 0.520 | 0.001 |  | 0.00 | | <0.01 | | | 0.00 - 0.00 | <-0.01 | 0.315 | 0.511 | -0.004 | |
| Occupational CLE → em | | 0.00 | <0.01 | -0.01 - 0.01 | <-0.01 | 0.792 | 0.876 | -0.006 |  | 0.00 | | <0.01 | | | -0.01 - 0.00 | <-0.01 | 0.814 | 0.873 | -0.004 | |
| Occupational CLE → sm | | 0.00 | 0.00 | 0.00 - 0.00 | 0.00 | 0.727 | 0.847 | -0.004 |  | 0.00 | | 0.00 | | | 0.00 - 0.00 | <0.01 | 0.247 | 0.457 | 0.004 | |
| Health-related CLE → cm | | 0.00 | <0.01 | 0.00 - 0.00 | <-0.01 | 0.157 | 0.310 | <.001 |  | 0.00 | | 0.00 | | | 0.00 - 0.00 | <-0.01 | 0.181 | 0.356 | -0.010 | |
| Health-related CLE → em | | 0.00 | <0.01 | 0.00 - 0.01 | 0.02 | 0.133 | 0.298 | 0.057 |  | 0.00 | | <0.01 | | | 0.00 - 0.01 | 0.01 | 0.148 | 0.323 | 0.047 | |
| Health-related CLE → sm | | 0.00 | 0.00 | 0.00 - 0.00 | 0.00 | 0.930 | 0.936 | -0.010 |  | 0.00 | | 0.00 | | | 0.00 - 0.00 | 0.00 | 0.838 | 0.873 | -0.001 | |
| Social CLE → cm | | 0.00 | <0.01 | 0.00 - 0.00 | -0.01 | 0.028 | 0.108 | 0.002 |  | 0.00 | | <0.01 | | | 0.00 - 0.00 | <-0.01 | 0.052 | 0.177 | -0.014 | |
| Social CLE → em | | 0.01 | <0.01 | 0.00 - 0.01 | 0.02 | 0.034 | 0.125 | 0.063 |  | 0.00 | | <0.01 | | | 0.00 - 0.01 | 0.02 | 0.034 | 0.129 | 0.054 | |
| Social CLE → sm | | 0.00 | 0.00 | 0.00 - 0.00 | <0.01 | 0.699 | 0.847 | -0.015 |  | 0.00 | | 0.00 | | | 0.00 - 0.00 | <0.01 | 0.155 | 0.323 | 0.009 | |
| Emotional neglect→ cm | | 0.00 | 0.00 | 0.00 - 0.01 | 0.00 | 0.185 | 0.347 | 0.004 |  | 0.00 | | 0.00 | | | 0.00 - 0.00 | 0.00 | 0.216 | 0.405 | 0.004 | |
| Emotional neglect→ em | | 0.01 | 0.01 | -0.01 - 0.02 | 0.01 | 0.290 | 0.493 | 0.016 |  | 0.00 | | 0.00 | | | 0.00 - 0.01 | 0.01 | 0.305 | 0.503 | 0.013 | |
| Emotional neglect→ sm | | 0.00 | 0.00 | 0.00 - 0.00 | 0.00 | 0.697 | 0.847 | -0.001 |  | 0.00 | | 0.00 | | | 0.00 - 0.00 | 0.00 | 0.159 | 0.323 | -0.005 | |
| Emotional abuse → cm | | 0.00 | 0.00 | 0.00 - 0.00 | 0.00 | 0.726 | 0.847 | -0.001 |  | 0.00 | | <0.01 | | | 0.00 - 0.00 | <-0.01 | 0.736 | 0.836 | -0.001 | |
| Emotional abuse → em | | 0.01 | 0.01 | 0.01 - 0.03 | 0.03 | 0.005 | 0.033 | 0.057 |  | 0.00 | | 0.00 | | | 0.01 - 0.02 | 0.02 | 0.008 | 0.044 | 0.048 | |
| Emotional abuse → sm | | 0.00 | 0.00 | 0.00 - 0.00 | 0.00 | 0.753 | 0.850 | 0.001 |  | 0.00 | | <0.01 | | | 0.00 - 0.00 | 0.00 | 0.296 | 0.492 | 0.003 | |
| Physical neglect → cm | | 0.00 | <0.01 | 0.00 - 0.00 | 0.00 | 0.550 | 0.767 | -0.001 |  | 0.00 | | <0.01 | | | 0.00 - 0.00 | <0.01 | 0.563 | 0.767 | 0.002 | |
| Physical neglect → em | | 0.03 | 0.01 | -0.01 - 0.01 | -0.01 | 0.653 | 0.841 | -0.008 |  | 0.02 | | <0.01 | | | -0.01 - 0.01 | <-0.01 | 0.642 | 0.810 | -0.008 | |
| Physical neglect → sm | | 0.00 | <0.01 | 0.00 - 0.00 | 0.00 | 0.726 | 0.847 | -0.001 |  | 0.00 | | <0.01 | | | 0.00 - 0.00 | <-0.01 | 0.255 | 0.463 | -0.003 | |
| Physical abuse→ cm | | 0.00 | 0.00 | 0.00 - 0.00 | 0.00 | 0.553 | 0.767 | -0.002 |  | 0.00 | | 0.00 | | | 0.00 - 0.00 | 0.00 | 0.568 | 0.767 | -0.002 | |
| Physical abuse→ em | | 0.00 | 0.01 | -0.01 - 0.02 | 0.01 | 0.449 | 0.706 | 0.010 |  | 0.00 | | 0.01 | | | -0.01 - 0.01 | 0.01 | 0.434 | 0.667 | 0.009 | |
| Physical abuse→ sm | | 0.00 | 0.00 | 0.00 - 0.00 | 0.00 | 0.737 | 0.847 | <.001 |  | 0.00 | | 0.00 | | | 0.00 - 0.00 | 0.00 | 0.279 | 0.485 | 0.002 | |
| Sexual abuse → cm | | 0.00 | <0.01 | 0.00 - 0.01 | <0.01 | 0.123 | 0.298 | 0.009 |  | 0.00 | | <0.01 | | | 0.00 - 0.00 | 0.00 | 0.138 | 0.319 | 0.008 | |
| Sexual abuse → em | | 0.01 | 0.01 | 0.02 - 0.04 | 0.08 | <.001 | <.001 | 0.196 |  | 0.01 | | <0.01 | | | 0.01 - 0.03 | 0.06 | <.001 | <.001 | 0.162 | |
| Sexual abuse → sm | | 0.00 | 0.00 | 0.00 - 0.00 | 0.00 | 0.710 | 0.847 | -0.001 |  | 0.00 | | <0.01 | | | 0.00 - 0.00 | <-0.01 | 0.217 | 0.405 | -0.006 | |
| Discrimination→ cm | | 0.00 | <0.01 | 0.00 - 0.00 | <0.01 | 0.171 | 0.327 | 0.004 |  | 0.00 | | <0.01 | | | 0.00 - 0.00 | <0.01 | 0.193 | 0.371 | 0.003 | |
| Discrimination→ em | | 0.02 | 0.01 | 0.01 - 0.01 | 0.07 | <.001 | <.001 | 0.073 |  | 0.01 | | <0.01 | | | 0.01 - 0.01 | 0.05 | <.001 | <.001 | 0.060 | |
| Discrimination→ sm | | 0.00 | 0.00 | 0.00 - 0.00 | 0.00 | 0.855 | 0.876 | <.001 |  | 0.00 | | <0.01 | | | 0.00 - 0.00 | <0.01 | 0.636 | 0.808 | 0.001 | |
| Social exclusion → cm | | 0.00 | 0.00 | 0.00 - 0.00 | 0.03 | <.001 | 0.002 | 0.042 |  | 0.00 | | <0.01 | | | 0.01 - 0.01 | 0.02 | 0.005 | 0.032 | 0.040 | |
| Social exclusion → em | | 0.00 | 0.00 | 0.01 - 0.01 | 0.18 | <.001 | <.001 | 0.313 |  | 0.00 | | <0.01 | | | 0.01 - 0.01 | 0.13 | <.001 | <.001 | 0.260 | |
| Social exclusion → sm | | 0.00 | <0.01 | 0.00 - 0.00 | 0.00 | 0.688 | 0.847 | -0.005 |  | 0.00 | | <0.01 | | | 0.00 - 0.00 | -0.01 | 0.105 | 0.297 | -0.025 | |
| PTSD event → cm | | 0.00 | <0.01 | 0.00 - 0.00 | <0.01 | 0.433 | 0.687 | <.001 |  | 0.00 | | <0.01 | | | 0.00 - 0.00 | <0.01 | 0.435 | 0.667 | 0.004 | |
| PTSD event → em | | 0.01 | <0.01 | 0.00 - 0.02 | 0.03 | 0.009 | 0.046 | 0.069 |  | 0.01 | | <0.01 | | | 0.00 - 0.01 | 0.02 | 0.010 | 0.052 | 0.058 | |
| PTSD event → sm | | 0.00 | 0.00 | 0.00 - 0.00 | 0.00 | 0.853 | 0.876 | 0.004 |  | 0.00 | | 0.00 | | | 0.00 - 0.00 | 0.00 | 0.637 | 0.808 | 0.001 | |

**S7. *R-Code***

## ===============================================================

## CFA & SEM (Mediation + Moderation)

## Project: JEPSY

## Notes:

## - Assumes a data.frame `df` exists with all variables below.

## - Indicators for mediators are harmonized upstream such that

## higher values indicate more adaptive functioning.

## - Includes EFA, baseline CFA, FINAL CFA with within-factor

## residual covariances (guided by MIs), and SEMs.

##

## ===============================================================

## ---------------- Parameters & setup ---------------------------

RUN_EFA <- TRUE

suppressPackageStartupMessages({

library(tidyverse)

library(lavaan)

library(semTools)

library(psych) # for EFA and KMO/Bartlett

})

## ---------------- Variable glossary (short) --------------------

# OUTCOMES (each modeled as a one-indicator latent):

# - J_LGcdrisc_Score : Resilience

# - J_LQzufrC10 : Life satisfaction

# - J_LQzufr17 : Meaning in life

# - J_ScoreInt : Internalizing symptoms

# - J_ScoreExt : Externalizing symptoms

# - J_ScoreSub : Substance use

#

# PREDICTORS (exposures; expected coding in parentheses):

# - J_PKptbs : Potentially traumatic events experienced (0/1; 1 = yes)

# - J_SFcts1_kat .. _5_kat : Childhood trauma indicators (0/1; 1 = yes)

# - J_SFdis : Discrimination (continuous; higher = more)

# - J_SFexklu : Social exclusion (continuous; higher = more)

# - J_BLevent_Soz : Major life events — Social domain (sum score; higher = more)

# - J_BLevent_Ber : Major life events — Occupational domain (sum score; higher = more)

# - J_BLevent_Ges : Major life events — Health domain (sum score; higher = more)

# - OSS3_Score : Perceived social support (continuous; moderator)

#

# MEDIATOR INDICATORS (higher = more adaptive; harmonized upstream):

# - Emotional process latent factor (“Emotional”):

# J_COPE_REP_rev, J_COPE_WIS_rev, J_SFpanas_Neg_rev, J_SFipsm_Score_rev

# - Cognitive process latent factor (“Cognitive”):

# J_SFerq_ScoreNB, J_COPE_PER, J_COPE_FLE, J_COPE_PRO

# - Social process latent factor (“Social”):

# J_SFerq_ScoreUD_rev, J_COPE_EMO, J_COPE_INS

#

# COVARIATES:

# - J_age, J_sex, J_SDbild1B_kat (education; dummies created).

## ---------------- Data presence & minimal checks ----------------

if (!exists("df") || !is.data.frame(df)) {

stop("Please provide a data.frame `df` with the variables listed in the glossary.")

}

required_vars <- c(

# outcomes

"J_LGcdrisc_Score","J_LQzufrC10","J_LQzufr17","J_ScoreInt","J_ScoreExt","J_ScoreSub",

# predictors & moderator (domain-level life events expected)

"J_PKptbs","J_SFcts1_kat","J_SFcts2_kat","J_SFcts3_kat","J_SFcts4_kat","J_SFcts5_kat",

"J_SFdis","J_SFexklu","J_BLevent_Soz","J_BLevent_Ber","J_BLevent_Ges","OSS3_Score",

# mediator indicators (harmonized; see glossary)

"J_COPE_REP_rev","J_COPE_WIS_rev","J_SFpanas_Neg_rev","J_SFipsm_Score_rev",

"J_SFerq_ScoreNB","J_COPE_PER","J_COPE_FLE","J_COPE_PRO",

"J_SFerq_ScoreUD_rev","J_COPE_EMO","J_COPE_INS",

# covariates

"J_age","J_sex","J_SDbild1B_kat"

)

missing_vars <- setdiff(required_vars, names(df))

if (length(missing_vars) > 0) {

stop("The following required variables are missing in `df`:\n",

paste(missing_vars, collapse = ", "), call. = FALSE)

}

## ---------------- Covariate harmonisation ----------------------

# Education dummies (reference = low)

df <- df %>%

mutate(

J_SDbild1B_mittel = as.integer(J_SDbild1B_kat == 2), #medium

J_SDbild1B_hoch = as.integer(J_SDbild1B_kat == 3), #high

J_SDbild1B_ausbildung = as.integer(J_SDbild1B_kat == 4) #in education

)

## ---------------- Moderator interactions (predictor × OSS3) ---

df <- df %>%

mutate(

mod_PKptbs = J_PKptbs * OSS3_Score,

mod_Soz = J_BLevent_Soz * OSS3_Score,

mod_Ber = J_BLevent_Ber * OSS3_Score,

mod_Ges = J_BLevent_Ges * OSS3_Score,

mod_cts1 = J_SFcts1_kat * OSS3_Score,

mod_cts2 = J_SFcts2_kat * OSS3_Score,

mod_cts3 = J_SFcts3_kat * OSS3_Score,

mod_cts4 = J_SFcts4_kat * OSS3_Score,

mod_cts5 = J_SFcts5_kat * OSS3_Score,

mod_dis = J_SFdis * OSS3_Score,

mod_exklu = J_SFexklu * OSS3_Score

)

## ---------------- EFA (exploratory factor analysis) ------------

# Goal: empirical check that mediator indicators align with a 3-factor

# structure (Emotional, Cognitive, Social). No interpretation beyond loadings.

if (RUN_EFA) {

med_efa <- df %>% select(

J_SFerq_ScoreNB, J_COPE_PER, J_COPE_FLE, J_COPE_PRO, # Cognitive

J_COPE_REP_rev, J_COPE_WIS_rev, J_SFpanas_Neg_rev, J_SFipsm_Score_rev,# Emotional

J_SFerq_ScoreUD_rev, J_COPE_EMO, J_COPE_INS # Social

)

print(psych::KMO(med_efa)) # KMO

print(psych::cortest.bartlett(med_efa)) # Bartlett

psych::fa.parallel(med_efa, fa = "fa", n.iter = 100)

efa3 <- psych::fa(med_efa, nfactors = 3, rotate = "oblimin")

print(efa3$loadings, cutoff = .30)

rm(efa3)

}

## ---------------- CFA (baseline) --------------------------------

cfa_model_baseline <- '

Cognitive =~ J_SFerq_ScoreNB + J_COPE_PER + J_COPE_FLE + J_COPE_PRO

Emotional =~ J_COPE_REP_rev + J_COPE_WIS_rev + J_SFpanas_Neg_rev + J_SFipsm_Score_rev

Social =~ J_SFerq_ScoreUD_rev + J_COPE_EMO + J_COPE_INS

'

fit_cfa_baseline <- lavaan::cfa(cfa_model_baseline, data = df,

estimator = "MLR", missing = "fiml")

# (Optional) inspect MIs guiding within-factor residual covariances

# mi <- modindices(fit_cfa_baseline, sort. = TRUE)

## ---------------- CFA (FINAL with within-factor covariances) ----

cfa_model_final <- '

# Latent factors

Cognitive =~ J_SFerq_ScoreNB + J_COPE_PER + J_COPE_FLE + J_COPE_PRO

Emotional =~ J_COPE_REP_rev + J_COPE_WIS_rev + J_SFpanas_Neg_rev + J_SFipsm_Score_rev

Social =~ J_SFerq_ScoreUD_rev + J_COPE_EMO + J_COPE_INS

# Within-factor residual covariances (Cognitive)

J_COPE_PER ~~ J_COPE_FLE

J_COPE_FLE ~~ J_COPE_PRO

J_SFerq_ScoreNB ~~ J_COPE_FLE

# Within-factor residual covariances (Emotional)

J_COPE_REP_rev ~~ J_COPE_WIS_rev

J_SFpanas_Neg_rev ~~ J_SFipsm_Score_rev

# Within-factor residual covariances (Social)

J_COPE_EMO ~~ J_COPE_INS

'

fit_cfa_final <- lavaan::cfa(cfa_model_final, data = df,

estimator = "MLR", missing = "fiml")

summary(fit_cfa_final, fit.measures = TRUE, standardized = TRUE)

## SEM: Mediation (three latent mediators) + moderation (interaction with social support)

## Outcome shown here: Resilience (latent). Repeat the same block for other outcomes.

# 1) Moderation terms: predictors × social support (OSS3_Score)

df <- df %>% mutate(

mod_PKptbs_OSS3 = J_PKptbs * OSS3_Score,

mod_BLevent_Soz_OSS3 = J_BLevent_Soz * OSS3_Score,

mod_BLevent_Ber_OSS3 = J_BLevent_Ber * OSS3_Score,

mod_BLevent_Ges_OSS3 = J_BLevent_Ges * OSS3_Score,

mod_cts1_OSS3 = J_SFcts1_kat * OSS3_Score,

mod_cts2_OSS3 = J_SFcts2_kat * OSS3_Score,

mod_cts3_OSS3 = J_SFcts3_kat * OSS3_Score,

mod_cts4_OSS3 = J_SFcts4_kat * OSS3_Score,

mod_cts5_OSS3 = J_SFcts5_kat * OSS3_Score,

mod_dis_OSS3 = J_SFdis * OSS3_Score,

mod_exklu_OSS3 = J_SFexklu * OSS3_Score

)

# 2) Model core: latent mediators, latent outcome, direct/indirect paths, covariates

sem_model_core <- '

##########################

## LATENT CONSTRUCTS ##

##########################

Emotional =~ J_COPE_REP_rev + J_COPE_WIS_rev + J_SFpanas_Neg_rev + J_SFipsm_Score_rev

Cognitive =~ J_SFerq_ScoreNB + J_COPE_PER + J_COPE_FLE + J_COPE_PRO

Social =~ J_SFerq_ScoreUD_rev + J_COPE_EMO + J_COPE_INS

Resilience =~ J_LGcdrisc_Score # latent outcome

############################################

## DIRECT (and moderated) PATHS TO OUTCOME

############################################

Resilience ~

c1*Emotional + c2*Cognitive + c3*Social +

c4*J_PKptbs + c4m*mod_PKptbs_OSS3 +

c5*J_BLevent_Soz + c5m*mod_BLevent_Soz_OSS3 +

c6*J_BLevent_Ber + c6m*mod_BLevent_Ber_OSS3 +

c7*J_BLevent_Ges + c7m*mod_BLevent_Ges_OSS3 +

c8*J_SFcts1_kat + c8m*mod_cts1_OSS3 +

c9*J_SFcts2_kat + c9m*mod_cts2_OSS3 +

c10*J_SFcts3_kat + c10m*mod_cts3_OSS3 +

c11*J_SFcts4_kat + c11m*mod_cts4_OSS3 +

c12*J_SFcts5_kat + c12m*mod_cts5_OSS3 +

c13*J_SFdis + c13m*mod_dis_OSS3 +

c14*J_SFexklu + c14m*mod_exklu_OSS3 +

J_age + J_sex +

J_SDbild1B_mittel + J_SDbild1B_hoch + J_SDbild1B_ausbildung

############################################

## PREDICTOR → MEDIATOR PATHS

############################################

Emotional ~

a1*J_PKptbs + a2*J_BLevent_Soz + a3*J_BLevent_Ber + a4*J_BLevent_Ges +

a5*J_SFcts1_kat + a6*J_SFcts2_kat + a7*J_SFcts3_kat +

a8*J_SFcts4_kat + a9*J_SFcts5_kat +

a10*J_SFdis + a11*J_SFexklu +

J_age + J_sex +

J_SDbild1B_mittel + J_SDbild1B_hoch + J_SDbild1B_ausbildung

Cognitive ~

b1*J_PKptbs + b2*J_BLevent_Soz + b3*J_BLevent_Ber + b4*J_BLevent_Ges +

b5*J_SFcts1_kat + b6*J_SFcts2_kat + b7*J_SFcts3_kat +

b8*J_SFcts4_kat + b9*J_SFcts5_kat +

b10*J_SFdis + b11*J_SFexklu +

J_age + J_sex +

J_SDbild1B_mittel + J_SDbild1B_hoch + J_SDbild1B_ausbildung

Social ~

d1*J_PKptbs + d2*J_BLevent_Soz + d3*J_BLevent_Ber + d4*J_BLevent_Ges +

d5*J_SFcts1_kat + d6*J_SFcts2_kat + d7*J_SFcts3_kat +

d8*J_SFcts4_kat + d9*J_SFcts5_kat +

d10*J_SFdis + d11*J_SFexklu +

J_age + J_sex +

J_SDbild1B_mittel + J_SDbild1B_hoch + J_SDbild1B_ausbildung

############################################

## SPECIFIC INDIRECT EFFECTS (labels match a*/b*/d* and c*)

############################################

trauma_emotion := a1 * c1

trauma_cognitive := b1 * c2

trauma_social := d1 * c3

blevent_sz_emotion := a2 * c1

blevent_br_emotion := a3 * c1

blevent_gs_emotion := a4 * c1

blevent_sz_cognitive:= b2 * c2

blevent_br_cognitive:= b3 * c2

blevent_gs_cognitive:= b4 * c2

blevent_sz_social := d2 * c3

blevent_br_social := d3 * c3

blevent_gs_social := d4 * c3

cts1_emotion := a5 * c1

cts2_emotion := a6 * c1

cts3_emotion := a7 * c1

cts4_emotion := a8 * c1

cts5_emotion := a9 * c1

cts1_cognitive := b5 * c2

cts2_cognitive := b6 * c2

cts3_cognitive := b7 * c2

cts4_cognitive := b8 * c2

cts5_cognitive := b9 * c2

cts1_social := d5 * c3

cts2_social := d6 * c3

cts3_social := d7 * c3

cts4_social := d8 * c3

cts5_social := d9 * c3

dis_emotion := a10 * c1

dis_cognitive := b10 * c2

dis_social := d10 * c3

exklu_emotion := a11 * c1

exklu_cognitive := b11 * c2

exklu_social := d11 * c3

'

# 3) Total effects (direct + sum of specific indirects)

lab_map <- tibble(

direct_lab = c("c4","c5","c6","c7","c8","c9","c10","c11","c12","c13","c14"),

ind_pref = c("trauma","blevent_sz","blevent_br","blevent_gs",

"cts1","cts2","cts3","cts4","cts5","dis","exklu")

) |>

mutate(ind_labels = map(ind_pref, ~ str_c(.x, c("_emotion","_cognitive","_social"), collapse = " + ")),

total_line = str_glue("{ind_pref}_total := {direct_lab} + {ind_labels}"))

sem_model <- paste(

sem_model_core,

"\n############################################\n## TOTAL EFFECTS\n############################################\n",

str_c(lab_map$total_line, collapse = "\n"),

sep = ""

)

# 4) Estimation:

fit <- lavaan::sem(

sem_model,

data = df,

estimator = "MLR",

se = "bootstrap",

test = "standard",

bootstrap = 20

)

# 5) Parameter table with bootstrap CIs, plus standardized (std.all)

pe <- parameterEstimates(

fit, ci = TRUE, level = .95, boot.ci.type = "perc",

standardized = FALSE, remove.system.eq = TRUE

) %>% mutate(rhs = coalesce(rhs, ""))

std_vals <- standardizedSolution(fit) %>%

rename(std.all = est.std) %>%

select(lhs, op, rhs, std.all)

pe2 <- pe %>% left_join(std_vals, by = c("lhs","op","rhs"))

# 6) Tables: Direct (to outcome), Defined (specific indirects), and Totals

make_tbl <- function(data, predictor_col, lbl) {

data %>%

transmute(

Predictor = !!rlang::sym(predictor_col),

B = est,

Beta = std.all,

SE = se,

CI_lower = ci.lower,

CI_upper = ci.upper,

p = pvalue,

Effect = lbl

)

}

direct_tbl <- pe2 %>% filter(op == "~", lhs == "Resilience") %>% make_tbl("rhs", "Direct")

indirect_tbl <- pe2 %>% filter(op == ":=", !str_detect(lhs, "_total$")) %>% make_tbl("lhs", "Indirect")

total_tbl <- pe2 %>% filter(op == ":=", str_detect(lhs, "_total$")) %>% make_tbl("lhs", "Total")

effects_tbl <- bind_rows(direct_tbl, indirect_tbl, total_tbl) %>%

mutate(Effect = factor(Effect, levels = c("Direct","Indirect","Total"))) %>%

arrange(Effect, Predictor)

# 7) Optional summary table (rounded)

effects_tbl_rounded <- effects_tbl %>%

mutate(across(c(B, Beta, SE, CI_lower, CI_upper, p), ~ round(., 3)))

# Preview (comment out in final rendering if needed)

# kable(effects_tbl_rounded, caption = "Effects on Resilience: Bootstrap estimates and CIs")

# 8) Standardized specific indirect effects (point estimates)

# StdIndirect = Beta × SDx / SDy;

# dis/exklu are min–max normalized (0–1) before SD is taken to harmonize scales.

sds <- df %>%

mutate(

J_SFdis_norm = (J_SFdis - min(J_SFdis, na.rm = TRUE)) / (max(J_SFdis, na.rm = TRUE) - min(J_SFdis, na.rm = TRUE)),

J_SFexklu_norm = (J_SFexklu - min(J_SFexklu, na.rm = TRUE)) / (max(J_SFexklu, na.rm = TRUE) - min(J_SFexklu, na.rm = TRUE))

) %>%

summarise(across(

c(J_PKptbs, J_BLevent_Soz, J_BLevent_Ber, J_BLevent_Ges,

J_SFcts1_kat, J_SFcts2_kat, J_SFcts3_kat, J_SFcts4_kat, J_SFcts5_kat,

J_SFdis_norm, J_SFexklu_norm, J_LGcdrisc_Score),

sd, na.rm = TRUE

)) %>%

tidyr::pivot_longer(everything(), names_to = "var", values_to = "SD")

sd_y <- sds %>% filter(var == "J_LGcdrisc_Score") %>% pull(SD)

predictor_to_var <- tibble(

Pattern = c("trauma","blevent_sz","blevent_br","blevent_gs",

"cts1","cts2","cts3","cts4","cts5","dis","exklu"),

VarName = c("J_PKptbs","J_BLevent_Soz","J_BLevent_Ber","J_BLevent_Ges",

"J_SFcts1_kat","J_SFcts2_kat","J_SFcts3_kat","J_SFcts4_kat","J_SFcts5_kat",

"J_SFdis_norm","J_SFexklu_norm")

)

indirect_std <- effects_tbl %>%

filter(Effect == "Indirect") %>%

mutate(Pattern = stringr::str_extract(Predictor, paste(predictor_to_var$Pattern, collapse = "|"))) %>%

left_join(predictor_to_var, by = "Pattern") %>%

left_join(sds, by = c("VarName" = "var")) %>%

mutate(Std_Indirect = round(as.numeric(Beta) * SD / sd_y, 4)) %>%

select(Predictor, Std_Indirect)

effects_tbl_with_std <- effects_tbl %>%

left_join(indirect_std, by = "Predictor") %>%

mutate(across(c(B, Beta, SE, CI_lower, CI_upper, p), ~ round(., 3)))

# Final table object for export (Direct/Indirect/Total; Indirect rows also carry Std_Indirect)

effects_tbl_with_std

# ------------------------------------------------------------

# 9) BH/FDR-Correction (Benjamini–Hochberg)

bh_tbl <- effects_tbl %>%

mutate(

# Assign each row to exactly one BH "family" (set of tests corrected together).

family = case_when(

Effect == "Indirect" ~ "Indirect",

Effect == "Direct" & str_detect(Predictor, "^mod_") ~ "Moderation",

Effect == "Direct" ~ "Direct",

TRUE ~ NA_character_

)

) %>%

group_by(family) %>%

# Compute BH-adjusted p-values within each family:

# p.adjust() implements the BH algorithm (steps 1–3 above).

mutate(

p_bh = ifelse(!is.na(family) & !is.na(p), p.adjust(p, method = "BH"), NA_real_)

) %>%

ungroup() %>%

select(Predictor, Effect, p_bh)

# Join BH-adjusted p-values back into the final table that already contains

# effect estimates, bootstrap CIs, and standardized indirect effects.

effects_tbl_with_std_bh <- effects_tbl_with_std %>%

left_join(bh_tbl, by = c("Predictor", "Effect"))

effects_tbl_with_std_bh_print <- effects_tbl_with_std_bh %>%

mutate(

p_raw = format.pval(p, digits = 3, eps = 1e-4),

p_BH = ifelse(is.na(p_bh), "—", format.pval(p_bh, digits = 3, eps = 1e-4)),

p_BH = ifelse(!is.na(p_bh) & p_bh < 0.05, paste0("**", p_BH, "**"), p_BH)

) %>%

select(Effect, Predictor, B, Beta, SE, CI_lower, CI_upper, p_raw, p_BH, Std_Indirect) %>%

rename(`p (raw)` = p_raw, `p (BH)` = p_BH)

knitr::kable(

effects_tbl_with_std_bh_print,

caption = "Effects on Resilience: Direct, indirect, and total effects with 95% bootstrap confidence intervals. BH-adjusted p-values are shown for Direct, Indirect, and Moderation effects (separately by effect type); Total effects are not BH-adjusted (—).",

escape = FALSE

)

------------------------------------------------------------------------
